# High-throughput cultivation and isolation of environmental anaerobes using selectively permeable hydrogel capsules

**DOI:** 10.1093/ismeco/ycaf117

**Published:** 2025-07-13

**Authors:** Hugo Sallet, Marion Calvo, Matteo Titus, Nicolas Jacquemin, Karin Lederballe Meibom, Rizlan Bernier-Latmani

**Affiliations:** Ecole Polytechnique Federale de Lausanne (EPFL), Environmental Microbiology Laboratory, CH-1015 Lausanne; Ecole Polytechnique Federale de Lausanne (EPFL), Environmental Microbiology Laboratory, CH-1015 Lausanne; Ecole Polytechnique Federale de Lausanne (EPFL), Environmental Microbiology Laboratory, CH-1015 Lausanne; Ecole Polytechnique Federale de Lausanne (EPFL), Environmental Microbiology Laboratory, CH-1015 Lausanne; Ecole Polytechnique Federale de Lausanne (EPFL), Environmental Microbiology Laboratory, CH-1015 Lausanne; Ecole Polytechnique Federale de Lausanne (EPFL), Environmental Microbiology Laboratory, CH-1015 Lausanne

**Keywords:** isolation, anaerobic microorganisms, hydrogel capsules, microfluidics, soil microbiome, methanogens, sulfate-reducing bacteria, acetogenic bacteria

## Abstract

Over the past two decades, metagenomics has greatly expanded our understanding of microbial phylogenetic and metabolic diversity. However, most microbial taxa remain uncultured, hindering research and biotechnological applications. Isolating environmental anaerobes using traditional methods is particularly cumbersome and low throughput. Here, we present a novel, high-throughput approach for the cultivation and isolation of anaerobes, which involves trapping and growing single microbes within selectively permeable hydrogel capsules followed by fluorescence-activated cell sorting to distribute compartmentalized isolates into liquid medium for further growth. We show that diverse anaerobes can grow within capsules and that slower-growing ones (e.g. methanogens) can be enriched with this platform. We also applied this approach to isolate anaerobes from soil, including strains of the sulfate-reducing bacteria *Desulfovibrio desulfuricans* and *Nitratidesulfovibrio vulgaris*. Overall, this work introduces a robust, high-throughput alternative to traditional techniques for isolating environmental anaerobes and expands the emerging set of microfluidics-based tools for the cultivation of novel taxa.

## Introduction

While cultivation-independent methods are now a gold standard for exploring microbiomes, the isolation of microorganisms remains imperative for studying their physiology, ecology, and evolution [1]. Cultivating individual strains in the laboratory offers numerous advantages: specific metabolisms (e.g. pollutant degradation, antibiotic synthesis) can be exploited, hypotheses (e.g. genome-based predictions) can be tested experimentally under controlled conditions, and high-quality genomes can be obtained to expand and enhance current databases.

However, our ability to isolate and cultivate microorganisms is extremely limited: it is estimated that less than 3% of prokaryotic species have been successfully cultivated [2–4], and around 90% of these are affiliated with only four phyla (Pseudomonadota, Bacillota, Actinomycetota, and Bacteroidota) [5, 6]. These numbers vary considerably across habitats: while cultured taxa predominate in human-associated microbiomes, most taxa found in underexplored environments (e.g. soil, terrestrial subsurface, hydrothermal vents) remain uncultured [7]. The discrepancy between the number of microorganisms observed under a microscope and those cultured using traditional methods (typically, agar plating), known as the “Great Plate Count Anomaly” [8], highlights our inability to mimic the complex conditions necessary for microbial growth.

Growth requirements can vary largely across taxa, and include not only suitable environmental conditions (e.g. temperature, pH, redox conditions) but also specific growth factors which are generally unknown [1]. In fact, even when these conditions are fulfilled, some microorganisms may not be able to grow because they are inhibited by others (e.g. competition for nutrients) or, in contrast, because they need others to grow (e.g. syntrophy, episymbiosis) [1]. Finally, conventional culture media often contain high concentrations of organic substrates and thus favour fast-growing taxa, which challenges the efforts to enrich and isolate slow-growers (e.g. oligotrophs) [9].

Efforts to improve “cultivability” rates by modifying artificial nutrient media have met limited success [10–12]. Culturomics approaches, which combine the diversification of culture conditions with systematic isolate screening (e.g. MALDI-TOF, colony imaging), have allowed the isolation of hundreds of novel taxa [13–15], but remain laborious and have only scarcely been applied to non-human microbiomes. Additionally, such approaches based on agar plating are ill-suited to cultivate environmental anaerobes [16, 17], some of which are key isolation targets (e.g. methanogens, anammox bacteria) [1, 18]. In particular, some obligate anaerobes (i.e. unable to utilize oxygen as a terminal electron acceptor) grow poorly at gas–liquid interfaces and require specific gases as energy or carbon sources. Alternative approaches, such as the roll-tube and the dilution-to-extinction methods [17, 19–21], have been employed to cultivate anaerobes but they are time-consuming and do not lend themselves to screening large numbers of isolates.

Within the past two decades, microfluidics-based methods have been developed to raise the throughput rate and facilitate the cultivation of low-abundance taxa. For instance, Ma et al. employed a custom microfluidic device (SlipChip) to concurrently perform genetic screening and cultivation of isolates in a high-throughput manner [22], enabling the targeted isolation of a novel anaerobic bacterium (member of Ruminococcaceae) from the human gut [23]. Another notable example is the iChip platform designed as an array of miniature diffusion chambers (microcompartments filled with microbial inoculum in agar-based medium and sealed with porous membranes), which advantageously allows for in situ cultivation, thus providing organisms with key environmental growth factors [24]. Although these platforms have aided the discovery of novel taxa, they have been used rarely since, possibly because of the difficulty to reliably craft and manipulate such miniature devices.

On the other hand, the increasing availability of fluorescence-activated cell sorting (FACS) has encouraged the development of isolation strategies based on trapping and growing single microorganisms in picolitre-size compartments, sorting these compartments based on the biomass enclosed within, and distributing these individually in medium to further grow the isolates. For instance, Zengler et al. have isolated novel strains from seawater and soil by cultivating microorganisms within agarose beads (i.e. permeable gel compartments), sorting and re-growing in microwell plates [25]. Recently, McCully et al. used a double-emulsion (i.e. water-in-oil-in-water) platform to grow stool-derived microorganisms. They observed an enrichment of slow-growing taxa (e.g. the Negativicutes Phascolarctobacterium faecium) – which they attributed to limited competition across compartments stemming from the low water–oil permeability [26] – though none of these taxa could be isolated using FACS.

Over the past decade, hydrogel capsules, i.e., microcompartments comprising a liquid core and a solid shell, have been increasingly popular in drug delivery and single-cell assays due to their robustness, biocompatibility and selective permeability [27–29]. Additionally, microfluidic instruments and reagent kits for capsule generation have become commercially available [30], making this technology accessible to microbiologists with no prior technical experience. Yet, although capsules have been shown to be compatible with microbial growth, they have not been used for isolating microorganisms.

This proof-of-concept study demonstrates the potential of selectively permeable hydrogel capsules (hereafter capsules) as a platform for the cultivation and isolation of anaerobes. We show that capsules support the growth of diverse anaerobic taxa, including those which fail to form colonies on agar. We also found that soil microorganisms grown in capsules were more diverse than those grown on agar and within agarose beads, suggesting that capsules may be advantageous to isolate novel taxa. Finally, following in-capsule cultivation, FACS was used to isolate anaerobes (including sulfate-reducing bacteria, SRB) from soil in a high-throughput manner. This approach can be adapted to different sample types and growth conditions and may be compatible with in situ cultivation.

## Materials and methods

In this work, we trap single microorganisms in capsules and grow them within. First, we grow known isolates anaerobically within capsules to establish feasibility. Next, we do the same with soil-extracted microorganisms, which were also grown simultaneously in other cultivation platforms (liquid, agar, agarose beads, water-in-oil droplets) to compare the cultivation potential of the different methods. We also follow the temporal changes in a soil-derived, capsule-entrapped microbial community. Finally, following in-capsule cultivation, we use FACS to isolate anaerobes – either directly extracted from soil or from enrichment cultures. See summary in Fig. S1.

### Generation of capsules

**Figure 1:**
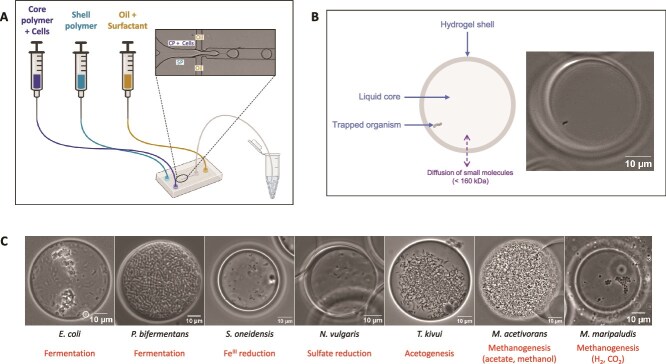
Single-cell encapsulation and growth of anaerobic microorganisms within capsules. (A) Overview of the capsule generation process (inset: microscope image of microfluidic chip during capsule generation). (B) Schematic and microscopy image showing a capsule enclosing a single bacterium (*Nitratidesulfovibrio vulgaris*). (C) Microscopy images of anaerobic strains growing within capsules. From left to right: *Escherichia coli* TB205 (18 h incubation), *Paraclostridium bifermentans* EML (24 h incubation), *Shewanella oneidensis* MR-1 (24 h incubation), *Nitratidesulfovibrio vulgaris* (2-day incubation), *Thermoanaerobacter kivui* (24 h incubation), *Methanosarcina acetivorans* C2A (18-day incubation) and *Methanococcus maripaludis* MM901 (5-day incubation). The type of anaerobic metabolism used by the strains is specified below each image. CP: Core polymer, SP: Shell polymer.

Capsules were produced with the Onyx instrument using the SPC Innovator Kit (CKN-G52, Atrandi Biosciences, Vilnius, Lithuania) [31]. Capsules are aqueous two-phase systems (ATPS) consisting of two immiscible aqueous phases, a core polymer and a shell polymer, combined in a microfluidic chip (Fig. 1A). While the former remains in the capsule core, the latter instantaneously migrates outwards upon encapsulation. Microbial cells were added to the core polymer immediately prior to encapsulation. Flow rates of 35 μl/h and 250 μl/h were used for the aqueous and oil phase, respectively. The encapsulation was monitored in real time with the high-speed camera of the Onyx instrument (Movie 1). The emulsion was briefly exposed to 405-nm light to induce crosslinking of the shell polymer, and subsequently broken as indicated in the supplier’s protocol (available upon request) [31]. This results in an aqueous suspension of 30–40 μm (14–33 pl) capsules (coefficient of variation ~15%), which are permeable to molecules smaller than 160 kDa (Fig. 1B). To maximize the encapsulation of single cells, the cell density in the aqueous phase was adjusted to 10^7^ cells/ml, achieving a Poisson parameter (λ) of 0.3. All steps were performed under an anoxic atmosphere (Fig. S2).

### Cultivation of known anaerobic strains in capsules

Seven phylogenetically and metabolically diverse anaerobic strains (Table 1) were individually compartmentalized into capsules, starting from fresh cultures. Following emulsion breaking, the capsules were resuspended in the appropriate media (Table 1) and incubated under anoxic conditions. Capsules were imaged under a microscope (Eclipse Ni-E, Nikon, Tokyo, Japan). Fluorescence imaging was also performed following a brief staining protocol (10-min incubation, 1 μM SYTO-9).

**Table 1:** Characteristics and cultivation conditions of microbial strains used in this study.

**Strain name**	**Source**	**Obligate/Facultative anaerobe**	**Metabolic group**	**Cultivation conditions** a
*Paraclostridium bifermentans* EML	Isolated from paddy soil [32]	Facultative	Fermenter	LB medium (BD Difco™), 30°C
*Shewanella oneidensis* MR-1	Lab collection	Facultative	Iron-reducing bacterium	M9 medium [33] amended with sodium lactate (20 mM) and ferric citrate (40 mM), 30°C
*Escherichia coli* TB205 (engineered by Bergmiller et al. [34])	Gift from Tobias Bergmiller	Facultative	Fermenter	LB medium (BD Difco™), 37°C
*Methanosarcina acetivorans* C2A	DSMZ (strain 2834)	Obligate	Methanogen	DSMZ 141c medium, N_2_/CO_2_ (80/20 v/v) headspace, 37°C
*Nitratidesulfovibrio vulgaris*	Lab collection	Obligate	Sulfate-reducing bacterium	DSMZ medium 63, 37°C
*Thermoanaerobacter kivui*	DSMZ (strain 2030)	Obligate	Homoacetogen	Complex medium described by Basen et al. [35], H_2_/CO_2_ (80/20 v/v) headspace, 60°C
*Methanococcus maripaludis* MM901 (engineered by Costa et al. [36])	Gift from Wenyu Gu	Obligate	Methanogen	DSMZ 141c medium, H_2_/CO_2_ (80/20 v/v) headspace, 37°C

a Media were brought to a boil, cooled down to room temperature under a gas flow (100% N_2_ or 80%,20% N_2_:CO_2_), and dispensed into 200-ml serum bottles (100 ml of medium per bottle) under the same gas atmosphere. The bottles were sealed with butyl rubber stoppers and aluminium crimps and autoclaved at 121°C for 30 min. Prior to autoclaving, the headspace of the serum bottles was flushed with the recommended gas.

### Soil sampling and extraction of soil microorganisms

Soil (upper 10 cm) was sampled from two adjacent rice paddies (A and B) in Mont-Vully, Switzerland (46°58′06.5″N 7°03′39.2″E), which were continuously flooded throughout the growing season (4 months). Samples were collected either 2 months after the harvest (A_AH_ and B_AH_) or shortly before the following year’s harvest (A_BH_ and B_BH_). Immediately after collection, the samples were placed in Mylar bags filled with argon and stored at 4°C. An aliquot of soil A_AH_ was stored at −80°C for DNA extraction. Soil physicochemical characteristics are described in Table S1.

To extract microorganisms, soil was mixed (1:4 volume) with a sodium pyrophosphate solution (0.2% in PBS) by vortexing and blending (30 s^−1^, 2 min) with a MM 400 mixer mill (Retsch, Haan, Germany). The resulting slurry was left to decant (30 min) and the supernatant was transferred onto a Nycodenz (Serumwerk Bernburg, Bernburg, Germany) solution (80% in PBS) at 4:1 volume ratio. After centrifugation (15 000 g for 90 min at 4°C), the cell-containing fraction was collected, washed several times in PBS and filtered (10 μm).

### Comparison of cultivation platforms for soil-derived microorganisms

Soil-extracted microorganisms were grown in capsules, in agarose beads, in water-in-oil droplets, in liquid, and on agar. Water-in-oil droplets (hereafter droplets) were generated with the Onyx platform using the Droplet Generation Kit (Atrandi Biosciences), with an aqueous phase composed of microbial cells suspended in medium. Agarose beads were generated as described by Schaerli [37]. For droplets and agarose beads, flow rates of 300 μl/h and 700 μl/h were used for the aqueous and oil phases, respectively. Cell density was adjusted as described above. Soil microorganisms were also spread on agar plates or directly inoculated in liquid medium. All steps were performed under an anoxic atmosphere and all cultivation platforms used the same Minimal Soil Medium (MSM10, see SI).

After incubation, microbial cells were extracted from capsules by dissolving the shell polymer with the Release Reagent (Atrandi Biosciences) at RT for 1 h under agitation (500 rpm). Agarose beads were dissolved by incubating with β-agarase I (5 units, 42°C, 30 min), following a first preheating step (65°C for 10 min). Capsules and agarose beads were washed twice in PBS before the extraction. Microorganisms were released from droplets by breaking the emulsion with 1*H*,1*H*,2*H*,2*H*-Perfluoro-1-octanol, as described previously [37]. Microbes grown on agar were harvested by adding PBS (2 ml) to agar plates, scraping the surface with an L-shaped spreader, and collecting the wash solution. Cells were collected from the liquid culture by centrifugation (8000 g, 5 min).

### Temporal change in composition of capsule-entrapped microbial community

Soil microorganisms were individually trapped in capsules and incubated in MSM10 medium for varying durations (24, 48, 72 and 144 hours) to evaluate the potential enrichment in slower-growing strains over time.

### Isolation of slow-growing soil anaerobes

The isolation of anaerobes was first attempted by encapsulating soil-extracted microorganisms and incubating the capsules either in MSM or in 10-ml serum bottles filled with enrichment media (DSMZ 135, DSMZ 311, or DSMZ 141c). Isolation was also attempted by encapsulating microorganisms from soil-derived cultures enriched in SRB and methanogens, using modified Postgate B and modified DSMZ 141c, respectively, as enrichment media. See SI for details. After growth, capsules were separated using FACS. Capsules were sorted with a FACSAria Fusion (BD Biosciences, Franklin States, NJ, USA), see details in SI. Prior to sorting, the capsules were washed three times in PBS and stained with SYTO-9 (2 μM, 15-min incubation), a fluorescent dye retaining cell viability. Two controls were prepared to facilitate the gating: one without SYTO-9 and another with SYTO-9 but incubated in PBS. More details on the gating are shown in Fig. S3. Single capsules were distributed into individual wells of 96-well plates filled with medium (200 μl/well) and incubated at 30°C in an anoxic chamber, or in a nox.18 anaerobic jar (SY-LAB, Purkersdorf, Austria) filled with H_2_ (80%) and CO_2_ (20%).

### DNA extraction and sequencing

The DNA from the soil-derived cultures was extracted with the PowerSoil Pro kit (Qiagen, Hilden, Germany) and concentrations were measured with a Qubit fluorometer (Invitrogen, Waltham, MA, USA). These samples were standardized to the same DNA concentration. Library preparation (Nextera DNA Flex, Illumina, San Diego, CA, USA) and sequencing (Novaseq 6000, Illumina, or Aviti, Element Biosciences) were performed at the Lausanne Genomic Technologies Facility (University of Lausanne, Switzerland).

The taxonomy of soil A_AH_ was profiled by full-length 16S rRNA gene sequencing, performed on a Sequel II (Pacific Biosciences, Menlo Park, CA, USA).

### Bioinformatic analysis

Shotgun metagenomic data were analyzed to profile the taxonomy of the cultures. Paired-end reads underwent quality control with fastp to remove adapters and low-quality reads, followed by an additional quality check with FASTQC. High-quality reads from individual samples were then independently assembled into contigs with MEGAHIT. Binning of assembled contigs was performed with BASALT, grouping contigs from all samples into one set of dereplicated metagenome-assembled genomes (MAGs). MAG quality was evaluated with Quast, and CheckM2 was used to further assess completeness and contamination. Only MAGs with >50% completeness and < 10% contamination were retained for downstream analysis. MAGs were taxonomically classified with GTDB-Tk default database. See SI for details.

### Taxonomic identification of isolates

Cell lysis was performed by mixing (1:4 v/v) the pure cultures with a Triton-X-100 buffer (0.1% v/v in TE) and heating at 99°C for 5 min. The lysates were spun down and the supernatant was used directly as DNA template in PCR reactions to amplify the near full-length 16S rRNA genes, using 27F (5′-AGA GTT TGA TCC TGG CTC AG-3′) and 1492R (5′-GGT TAC CTT GTT ACG ACT T-3′) primers. Further sample purification and Sanger sequencing were performed by Microsynth (Balgach, Switzerland).

## Results

Selectively permeable capsules are hydrogel microcompartments whose pore size is both small enough to retain cells and large enough to allow the diffusion of small biomolecules (Fig. 1B). In addition, they include a rigid shell, allowing physical separation from bulk solution, and a liquid core, allowing planktonic microbial growth, making them distinct from other types of microcompartments (droplets, agarose beads) (Fig. S4). We set out to leverage the recent commercial availability of this technology to provide a proof-of-concept for its use to cultivate and isolate environmental anaerobes.

### Capsules support the growth of known anaerobic strains

Seven anaerobic strains, varying in phylogeny, metabolism, and oxygen sensitivity (Table 1), were all able to grow within capsules (Fig. 1C). Fluorescence microscopy could successfully reveal microbial cells enclosed within capsules (Fig. S5), and their active metabolic state was evidenced by their motility (Movies 2–6). Interestingly, both a planktonic lifestyle and biofilm formation were observed within capsules containing E. coli (Fig. 1C, Movie 3). Therefore, diverse microbial species can grow anaerobically within capsules.

### Growth of distinct soil microbial taxa across cultivation platforms

We then examined whether soil microorganisms could grow anaerobically within capsules, and, if so, which taxa would preferentially grow in this cultivation platform compared to others, i.e. liquid, agar, agarose beads, droplets. The latter two represent compartmentalized platforms with characteristics distinct from capsules (Fig. S4).

The soil was dominated by microbial taxa in the phyla Bacillota (formerly Firmicutes), Pseudomonadota (formerly Proteobacteria) and Bacteroidota, and also contained members of Desulfobacterota (expected SRB) in substantial (6%) proportions (Fig. S6).

**Figure 2:**
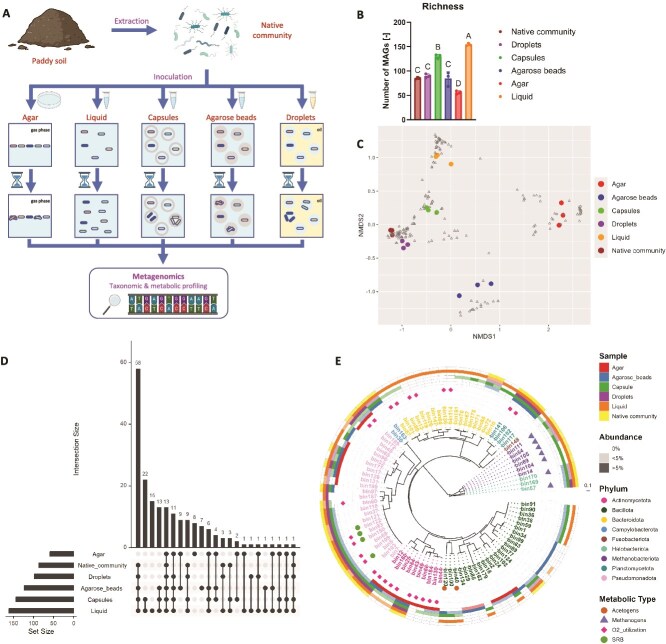
Comparison of different platforms to cultivate soil microbial taxa. (A) Schematic overview of the experiment. Microorganisms are extracted from a paddy soil (A_BH_) and grown anaerobically for 1 week in different cultivation platforms. After cultivation, microbial cells are harvested and all communities (initial and 1-week cultures) are profiled with metagenomics. (B) Richness of microbial communities. Bar lengths indicate mean richness values (number of MAGs) and error bars show standard deviation (N = 3 replicates). Individual replicate values are shown as dots. Different letters indicate significant difference at p < 0.05 between treatments (Tukey’s test). (C) Nonmetric multidimensional scaling (NMDS) plot illustrating differences across microbial communities, based on Bray–Curtis dissimilarity values derived from relative abundance data. The reads that did not map to any MAGs were removed from the analysis and relative abundance values were rescaled accordingly. Each MAG is shown as a triangle, whereas communities are depicted as colour dots. Different colours indicate different cultivation conditions (three replicates per condition). The stress value of the ordination was 0.0605. (D) UpSet plot showing taxonomic similarities across the different communities. The set size indicates, for each condition, the total number of MAGs who are present in at least one replicate. Intersection size shows the number of MAGs shared across the samples indicated with dots. (E) Circular maximum likelihood phylogenetic tree illustrating the diversity of microbial taxa (MAGs) present in the communities, based on the relative abundance values (average of 3 replicates). The colour of the MAG name indicates its taxonomy at the phylum level and its presence/absence in the different communities is shown in the outer circles (one specific colour is attributed to each sample type). Two abundance ranges (<5% and > 5%) are specified with different colour shades. Symbols indicate MAGs with specific metabolic capabilities (acetogens, methanogens, aerobes and sulfate-reducers). The scale represents the branch length. SRB: Sulfate-reducing bacteria.

Soil-extracted microbial cells were cultured individually in compartments (capsules, agarose beads, droplets), and concomitantly on solid (agar) and in liquid medium (Fig. 2A) and growth was observed microscopically within a few days (Fig. S7). After one week of growth, the highest richness (number of MAGs) was observed in the liquid culture (155 ± 2.6 MAGs), and the lowest on agar (57 ± 3.6 MAGs) (Fig. 2B). Within compartmentalized systems, capsules (131 ± 4.4 MAGs) showed higher richness than droplets (91 ± 4.0 MAGs) and agarose beads (85 ± 14.6 MAGs) (Fig. 2B). A strong deviation from the native community was observed in the agar, agarose beads, and liquid cultures (Fig. 2C), whose resulting communities were associated with 8, 7 and 15 unique signature MAGs, respectively (Fig. 2D). As expected, agar plating did not allow the growth of most microbial taxa (including 58 MAGs found in all other conditions) (Fig. 2D), and strongly favoured phyla associated with faster growth rates and aerobic metabolisms (Actinomycetota, Bacillota and Pseudomonadota) (Fig. 2E). On the other hand, capsule-grown microorganisms were more reflective of the original community structure and showed high similarity with liquid cultures (22 shared MAGs) (Fig. 2C-D). Notably, phyla associated with slow-growing taxa (Methanobacteriota, Halobacteriota, Fusobacteriota) were all found in liquid and capsules, and to a lesser extent in agarose beads and emulsion cultures, but none were detected in the agar cultures (Fig. 2E). In particular, seven taxa in the classes Methanobacteria (bin111, bin54, bin105, bin69, bin104) and Methanosarcinia (bin170, bin57) were identified as methanogens, and were present in the capsules (Fig. 2E, Table S2). Interestingly, poorly characterized taxa, such as *Anaerorhabdus* sp. (bin 162) and Fusobacteriaceae gen. sp. (bin 148), were only detected in liquid and capsules (Fig. 2E, Table S2). We also performed a similar analysis directly on the short-read sequences (using mOTUs) – to exclude potential biases caused by metagenomic assembly – and observed similar trends (Fig. S8). Interestingly, microorganisms within the class Clostridia were only shown to grow in liquid, agarose beads, and capsules (Fig. S9-S10). The mOTUs taxonomy also showed evidence of Desulfovibrionia taxa (putative SRB) growing in liquid and within capsules (Fig. S10). Collectively, these results indicate that a broad range of soil microorganisms, including poorly characterized taxa, can grow within capsules.

### Soil microbial community undergoes temporal shifts during in-capsule cultivation

**Figure 3:**
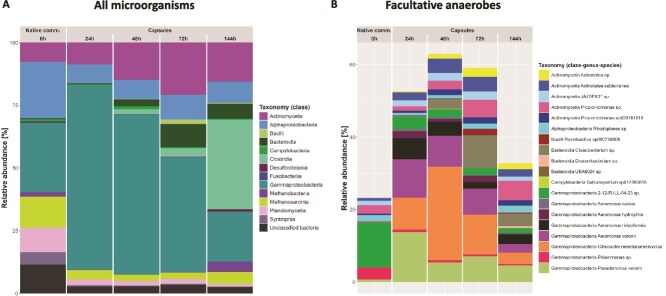
Dynamics of soil-derived microbial community within capsules over cultivation time. (A) Taxonomy barplot showing changes in the microbial community extracted from soil (A_BH_) throughout cultivation in capsules in MSM10 medium. The MAGs are shown at the class level (see colour code in legend). Taxa labelled as “unclassified bacteria” are MAGs that were not assigned to a known phylum. (B) Taxonomy barplot showing changes in the abundance of facultative anaerobic taxa throughout cultivation in capsules. The taxonomy (family, genus and species) of each MAG is indicated, see colour code in legend.

We cultivated soil microorganisms anaerobically within capsules and profiled the taxonomy over time. Gammaproteobacteria taxa grew rapidly and dominated the community within 24 h, but then decreased in relative abundance as other taxa (e.g. Actinomycetia, Clostridia, Bacteroidia) grew substantially between 24 h and 72 h (Fig. 3A). A closer look at the facultative anaerobic taxa (MAGs harbouring genes involved in oxygen respiration) showed a sharp increase in their relative abundance in the first 48 h, followed by a decrease, especially between 72 h and 144 h (Fig. 3B). The growth of strictly anaerobic taxa (Methanobacteria, Methanosarcinia and Desulfovibrionia) occurred during this time frame (Fig. 3A). In a parallel experiment where the spent medium was replaced with fresh medium after every time point, a similar result was observed (Fig. S11). We also attempted to further enrich slow-growing taxa by conducting similar experiments with longer incubation periods, but did not observe their growth (Fig. S12-S13). Therefore, individual taxa exhibit distinct in-capsule growth dynamics.

### High-throughput capsule sorting enables the isolation of soil anaerobes

Similar to agarose beads and double-emulsion systems [26, 37], we found that capsules can be sorted with FACS. Thus, we set out to isolate anaerobes using this high-throughput technique. Capsule-entrapped soil microorganisms were cultivated, the biomass fluorescently labeled and sorted into microwells to establish pure cultures. With this approach, we were able to isolate various soil-derived microorganisms, including the obligate anaerobe *Clostridium* J sp. 902363375 (Table 2).

**Table 2:** List of slow-growing anaerobes isolated from soil using capsules and FACS.

**Soil**	**Organisms in capsules**	**Medium (cultivation within capsules)** a	**Relative abundance within capsules at the time of sorting** b	**Medium (cultivation in microwell plate)** a	**Taxonomy of isolate** c	**Yield** d
A_AH_	Freshly extracted from soil	MSM1	Not measured	MSM10	*Clostridium* J sp. 902363375	1/25 (4.0%)
A_BH_	Freshly extracted from soil	DSMZ 311 medium, H_2_/CO_2_ (80/20 v/v, 0.5 bar)	0%	DSMZ 311 medium	*Terrisporobacter glycolicus*	1/68 (1.5%)
A_BH_	Freshly extracted from soil	DSMZ 135 medium, H_2_/CO_2_ (80/20 v/v, 0.5 bar)	8.4%	DSMZ 135 medium	*Acetoanaerobium sticklandii*	1/35 (2.9%)
A_BH_	Freshly extracted from soil	DSMZ 311 medium, H_2_/CO_2_ (80/20 v/v, 0.5 bar)	Not measured	DSMZ 311 medium	*Paraclostridium* sp.	1/24 (4.2%)
B_BH_	Enrichment culture (SRB)	Modified Postgate B medium, N_2_/CO_2_ (20/80 v/v, 0.5 bar)	Not measured	Modified Postgate B medium, H_2_/CO_2_ (20/80 v/v, 0.1 bar)	*Desulfovibrio desulfuricans*	1/151 (0.7%)
B_BH_	Enrichment culture (SRB)	Modified Postgate B medium, N_2_/CO_2_ (20/80 v/v, 0.5 bar)	Not measured	Modified Postgate B medium, H_2_/CO_2_ (20/80 v/v, 0.1 bar)	*Lacrimispora xylanolytica*	1/151 (0.7%)
B_BH_	Enrichment culture (methanogens)	Modified DSMZ 141c medium, H_2_/CO_2_ (80/20 v/v, 2 bar)	Not measured	DSMZ 141c medium, H_2_/CO_2_ (80/20 v/v, 0.1 bar)	*Nitratidesulfovibrio vulgaris*	2/112 (1.8%)

a Cultivation was performed at 30 °C. When no gases are specified, the cultures were incubated in an anoxic chamber (97% N_2_, 3% H_2_). More information about the media can be found in SI.

b An aliquot of the capsules was stored for DNA extraction 3 h before FACS. Relative abundance values were obtained after mOTUs taxonomic assignment.

c The taxonomy of each isolate was determined by comparing its near-full length 16S rRNA gene (PCR-amplified with the 27F-1492R primers) to the GTDB database (release 202).

d Ratio between the number of isolates of the corresponding taxon and the total numbers of isolates obtained under the same conditions.

**Figure 4:**
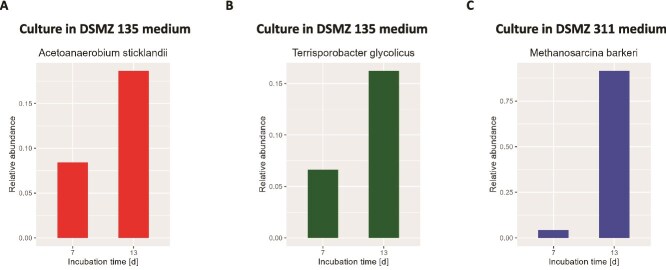
Growth of strictly anaerobic taxa within capsules in enrichment medium. Soil (A_BH_) microorganisms were individually entrapped in capsules and cultivated at 30°C in pre-reduced enrichment media (either DSMZ 135 or DSMZ 311 medium) in serum bottles filled with 80% H_2_, 20% CO_2_. After 7 and 13 days, an aliquot of the culture was sacrificed for taxonomic profiling (mOTUs). Barplots show the enrichment of (A) *Acetoanerobium sticklandii*, (B) *Terrisporobacter glycolicus* and (C) *Methanosarcina barkeri* within capsules, between 7 and 13 days of incubation. The data in (A) and (B) are from the same culture.

However, because most of the isolates obtained with the MSM medium were fast-growing, facultative anaerobic taxa (Fig. S14A), we repeated the experiment by using media containing reducing agents to promote slower-growing taxa. This approach proved successful, as we observed an enrichment of strict anaerobes within capsules (Fig. 4, Fig. S15), and could isolate some of them (*Terrisporobacter glycolicus*, *Acetoanaerobium sticklandii*, *Paraclostridium* sp.) (Table 2, Fig. S14B-D).

We further aimed to isolate SRB and methanogens, so we specifically enriched these microbial groups prior to encapsulation by incubating the soil under the appropriate selective conditions. This approach allowed us to isolate two SRB from soil (*Desulfovibrio desulfuricans* and *Nitratidesulfovibrio vulgaris*) (Table 2, Fig. S14E-F).

No methanogens were isolated from soil, although they were sometimes found in high abundance in capsules (Fig. 4C, Fig. S15). To assess the potential of our approach to isolate methanogens, we performed a control experiment where *Methanococcus maripaludis* was grown within capsules which were individually sorted in microwell plates. After a one-week incubation period, many wells showed growth of *M. maripaludis* (Fig. S16), while control wells (containing only medium) remained sterile. Thus, this strategy holds great potential to isolate diverse environmental anaerobes.

## Discussion

In this study, we presented a novel cultivation approach consisting of trapping and growing single microorganisms within capsules. We demonstrated the growth of phylogenetically and metabolically diverse anaerobes within capsules, including SRB, acetogens, and methanogens (Fig. 1C, 2E, 3A). Capsules act as microenvironments where microorganisms are physically separated but chemically interconnected. The selectively permeable shell of the capsule allows diffusion of nutrients and metabolites (including dissolved gases), enabling interspecies interactions (e.g. cross-feeding, competition). This likely explains why soil-derived cultures grown within capsules resemble batch liquid cultures (Fig. 2C-E).

However, capsules are compartments with limited space, restricting microbial growth to their maximum holding capacity. The decline in the growth of Gammaproteobacteria taxa following an early expansion phase may reflect the saturation of this available space (Fig. 3A-B), especially as medium replenishment did not allow further growth of these taxa (Fig. S11). Over a certain load, cell division may be inhibited by compressive forces or by a harmful accumulation of waste products. Therefore, in contrast with batch cultivation, capsules may advantageously prevent the proliferation of fast-growing microorganisms.

### Capsules enable the growth of diverse soil microbial taxa

Unlike agarose beads, which hold cells within a solid matrix, capsules allow microorganisms to grow as free-floating cells within a liquid core (Fig. S5). This distinction may explain the large differences between the cultures in these microcompartments (Fig. 2C-E, Fig. S9-S10). Certain taxa such as Fusobacteriaceae gen. sp. (bin148), *Anaerorhabdus* sp. (bin 162) and many members in the phylum Bacteroidota, were found to grow within capsules and in batch liquid cultures but not within agarose beads (Fig. 2E, Table S2). Microbial cultivation within agarose beads was strongly biased towards specific MAGs, in particular members of the classes Clostridia and Bacilli (Fig. S10-S11). This may indicate that spore-forming bacteria are better fitted to thrive within gel matrices. In addition, nine taxa grew exclusively on agar and within agarose beads (Fig. 2D), which suggests surface attachment improved their fitness. Such mechanisms have been observed in biofilm-forming bacteria through the upregulation of genes involved in stress response and virulence [38, 39]. Alternatively, these microorganisms may be able to enzymatically degrade agarose (also a major component of agar) and utilize it as source of carbon or energy [40].

Consistent with the Great Plate Count Anomaly, only a small fraction of soil microorganisms grew on agar (Fig. 2B). In particular, no archaea and no member of the phyla Bacteroidota, Planctomycetota and Fusobacteriota were found in the agar cultures, whereas Actinomycetota members were highly abundant (Fig. 2E). This bias toward facultative anaerobes may result from the presence of trace levels of oxygen at the gas–liquid interface or from the formation of hydrogen peroxide upon autoclaving phosphate and agar together [12, 41, 42].

Recently, McCully et al. stressed the importance of nutrient privatization for cultivating slow-growing taxa, some of which (Methanobacteria, Negativicutes) they enriched from human stool using a double-emulsion platform (water-in-oil-in-water) [26]. However, the authors also proposed that chemical diffusion may occur to a limited extent through the thin oil layer and allow the growth of certain microorganisms via cross-feeding. Here, although similar taxa (Methanobacteria, Methanosarcinia, Desulfovibrionia and Negativicutes) were observed within capsules and in batch liquid culture, where nutrient sharing and interspecies interactions (e.g. competition) take place, we have not evidenced their enrichment in single emulsion (droplets) (Fig. 2E, 3A, S9-S10). A greater nutrient privatization is expected in droplets as compared to double emulsion, because of the significantly thicker oil barrier separating aqueous compartments (i.e. chemical diffusion is slower). The preclusion of interspecies interactions may explain why some organisms, such as the hydrogenotrophic methanogen *Methanobacterium lacus* (bin69, bin104, and bin14), did not grow within droplets (Fig. 2E). Therefore, we propose that, under certain conditions, interspecies interactions are more important than nutrient privatization for enriching slow-growing taxa.

Positive interspecies interactions may have enabled the growth of certain taxa within capsules. For instance, we evidenced the growth of a novel species in the Negativicutes genus *Sporomusa* (bin120) (Fig. 2E, Table S2), whose members can perform acetogenesis and fermentation of *N*-methyl compounds [43]. Metagenomic data indeed reveal the presence of genes involved in the Wood-Ljungdahl pathway in this organism, thus requiring CO_2_ and H_2_ (Table S2) These growth substrates are likely produced by other organisms, typically fermenters, before being utilized by *Sporomusa* sp. We also evidenced the growth of methanogens harbouring genes for all known methanogenic pathways (Fig. 2E, Table S2). Methanogenic substrates (e.g. acetate, H_2_) may similarly be released by fermentative organisms, which would explain why the growth of methanogens occurs only after an increase in abundance of Bacteroidia and Clostridia taxa (Fig. 3A, S11). In other experiments, the lack of sufficient fermentative activity may be responsible for the inability of SRB and methanogens to grow (Fig. S12-S13). Finally, facultative anaerobes may set the stage for the growth of strict anaerobes by consuming residual oxygen, lowering the redox potential.

### Novel taxa grow in capsules

This study also revealed the presence of yet-uncultured but environmentally relevant taxa in soil-derived cultures. For instance, peculiar Burkholderiales bacteria (bin93, bin150 and bin5) were found with the potential to fully reduce sulfate to sulfide while also being capable of respiring oxygen (Fig. 2E, Table S2). Similar taxa had been reported in sulfate-rich zones within estuary sediments [44]; here, they may possibly occupy sulfate-rich soil microsites. Also, we identified microbial taxa in the candidate genus *Anammoximicrobium* (bin141, bin106 and bin182) (Fig. 2E, Table S2), i.e. putative anaerobic ammonium oxidizers, which had previously been observed in wastewater [45]. While these taxa were all present in capsule-grown cultures, none were detected in agar-grown cultures.

### Isolation of anaerobes using capsules

Capsules can be sorted individually to isolate microorganisms, including obligate anaerobes, in a high-throughput manner. This is of high interest because anaerobes are traditionally isolated with labour-intensive techniques (e.g. dilution-to-extinction, Hungate roll-tube). One advantage of FACS is that only a very low biomass (< 100 cells) is required for sorting. In comparison, conventional isolation methods based on visual screening of colonies may require millions of cells. We also found that, like agarose beads [37], capsules break open immediately after sorting, which conveniently removes the need for dissolving them before growing isolates in liquid medium.

The comparison across platforms showed that both capsules and droplets can effectively recapitulate the initial soil community (Fig. 2B-E). However, droplets cannot be readily separated from the oil phase (due to the absence of a rigid shell) and therefore, cannot be FACS sorted. Double-emulsion droplets (McCully et al., 2023) can be sorted but still suffer from the requirement to add the DNA stain upon encapsulation as opposed to after growth as for capsules.

Here, we successfully isolated soil anaerobes via capsule sorting (Table 2). The incubation of capsules in enrichment media prior to sorting allowed us to enrich (Fig. 4, Fig. S15) and isolate (Table 2) slow-growing microorganisms, including two putative acetogens, *Acetoanaerobium sticklandii* and *Terrisporobacter glycolicus* [46]. We also enriched for SRB and methanogens before encapsulation, which allowed us to isolate *D. desulfuricans* and *N. vulgaris*, two SRB. However, no methanogens were isolated, despite their ability to grow within capsules (Fig. 4C). Some methanogens are known to initiate growth only when the redox potential is sufficiently low (< 0 mV) [47, 48]. Since FACS was not performed under anoxic conditions, we suspect that this short exposure to air may have inhibited their growth. Yet, an isolation workflow conducted under similar conditions with the methanogen *M. maripaludis* resulted in its growth in the microwell plate (Fig. S16). Thus, soil methanogens may require specific growth factors released by other microorganisms which were not provided in our medium in the microplate.

In previous studies, microbial growth had been shown to occur within capsules but only with readily cultivable model organisms under aerobic conditions [27, 49, 50]. This work demonstrates that this platform can further be used to study unknown environmental taxa. For instance, single-cell compartmentalization, cultivation within capsules and shell dissolution steps may be repeatedly performed to enrich rare taxa. Several microorganisms may also be co-encapsulated to enrich elusive syntrophic taxa.

Capsules had, to our knowledge, not previously been used in combination with FACS to isolate microorganisms. Here, we demonstrated the strong potential of this approach by isolating slow-growing anaerobic bacteria from soil. In contrast, agarose beads and droplets have been employed for isolation, but we show that only a small subset of microbial taxa grow in these systems as compared to capsules.

We envision that in situ incubation of capsules prior to sorting could facilitate the isolation of novel taxa. Ben-Dov et al. have previously shown the potential of in situ cultivation by reporting the growth of novel coral-associated microorganisms within polysulfone-coated agar beads, although this has not resulted in isolation [51]. Here, capsules could be incubated in diffusion chambers directly placed into the native environment [52, 53]. The success of the isolation would depend on the ability of the organisms to grow in the microwell plate after FACS, which could be aided by the use of dedicated environment-mimicking media. Furthermore, capsules may facilitate the isolation of hyperthermophilic microorganisms for which agar, melting at 85°C, cannot be used [54]. Here we have shown the growth of a thermophile (*Thermoanaerobacter kivui*) at 60°C (Fig. 1C), but capsules can even stand heat up to 100°C [55].

## Supplementary Material

figS1_ycaf117

figS2_ycaf117

figS3_ycaf117

figS4_ycaf117

figS5_ycaf117

figS6_ycaf117

figS7_ycaf117

figS8_ycaf117

figS9_ycaf117

figS10_ycaf117

figS11_ycaf117

figS12_ycaf117

figS13_ycaf117

figS14_ycaf117

figS15_ycaf117

figS16_ycaf117

figS17_ycaf117

figS18_ycaf117

M1_ycaf117

M2_ycaf117

M3_ycaf117

M4_ycaf117

M5_ycaf117

M6_ycaf117

supplementary_information_and_captions

Table_S2_ycaf117

## Data Availability

All sequencing data are accessible under the NCBI BioProject PRJNA1190322. The code used in this study is available on GitHub.
