# Supplementary material for: High-throughput cultivation and isolation of environmental anaerobes using selectively permeable hydrogel capsules
Source: ISME Commun. 2025 Jul 13;5(1):ycaf117. doi: 10.1093/ismeco/ycaf117 (PMC12319321; doi:10.1093/ismeco/ycaf117)
Supplement: figS1_ycaf117 [file figs1_ycaf117.pdf]

Comparison of different cultivation platforms (Fig. 2)

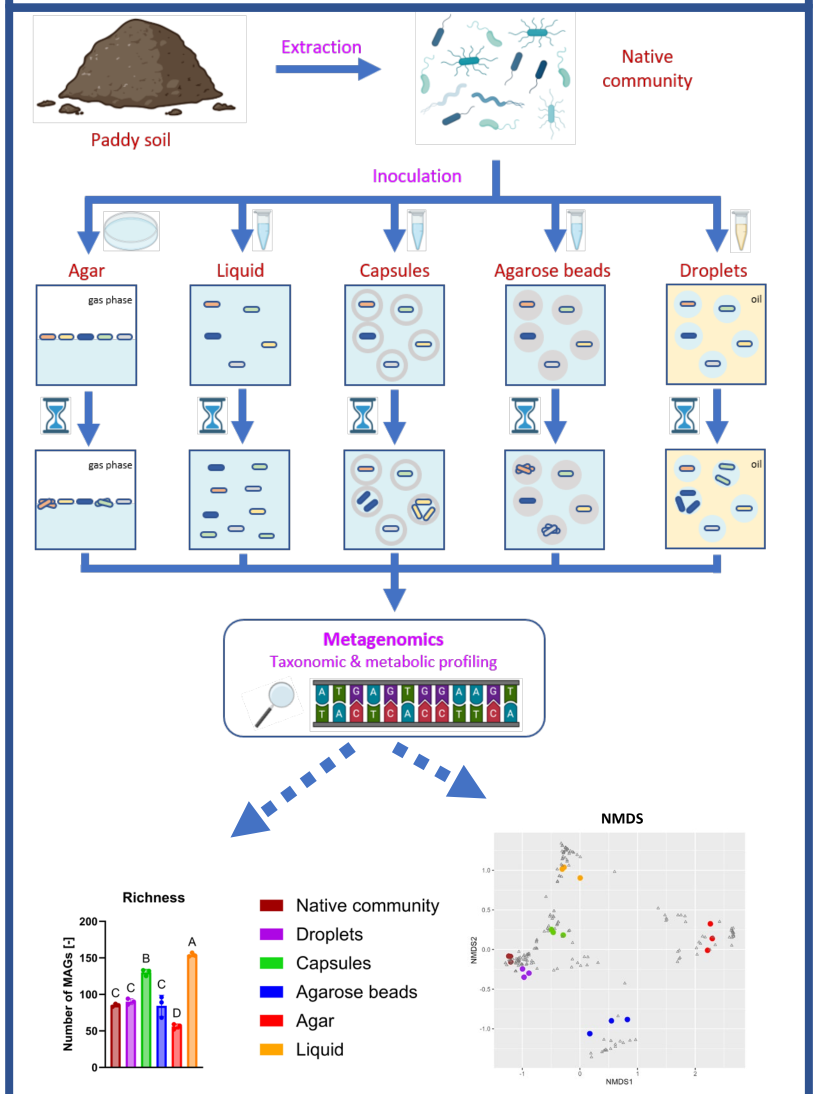

Soil microorganisms are grown in a minimal medium using different cultivation platforms. After incubation, the communities are compared.

Changes in microbial community over time in capsules (Fig. 3)

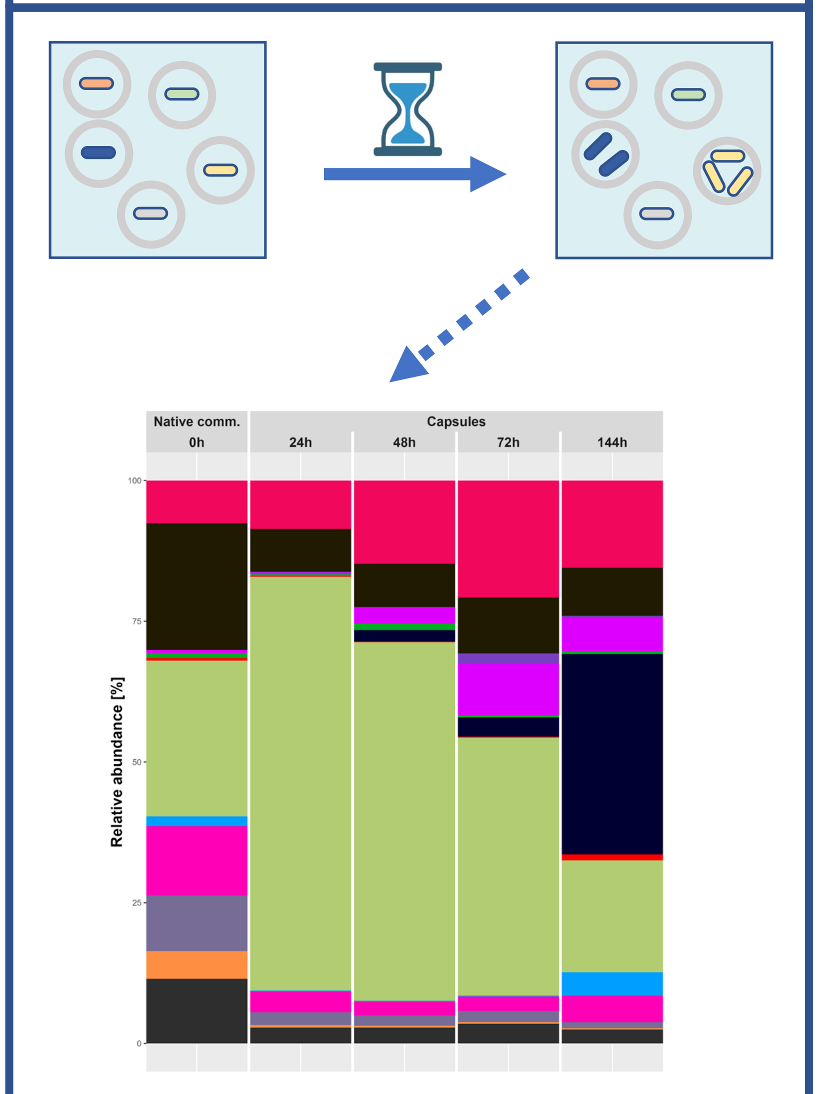

Soil microorganisms are grown in hydrogel capsules and the taxonomy is profiled after different incubation periods.

Isolation of anaerobes (Table 2)

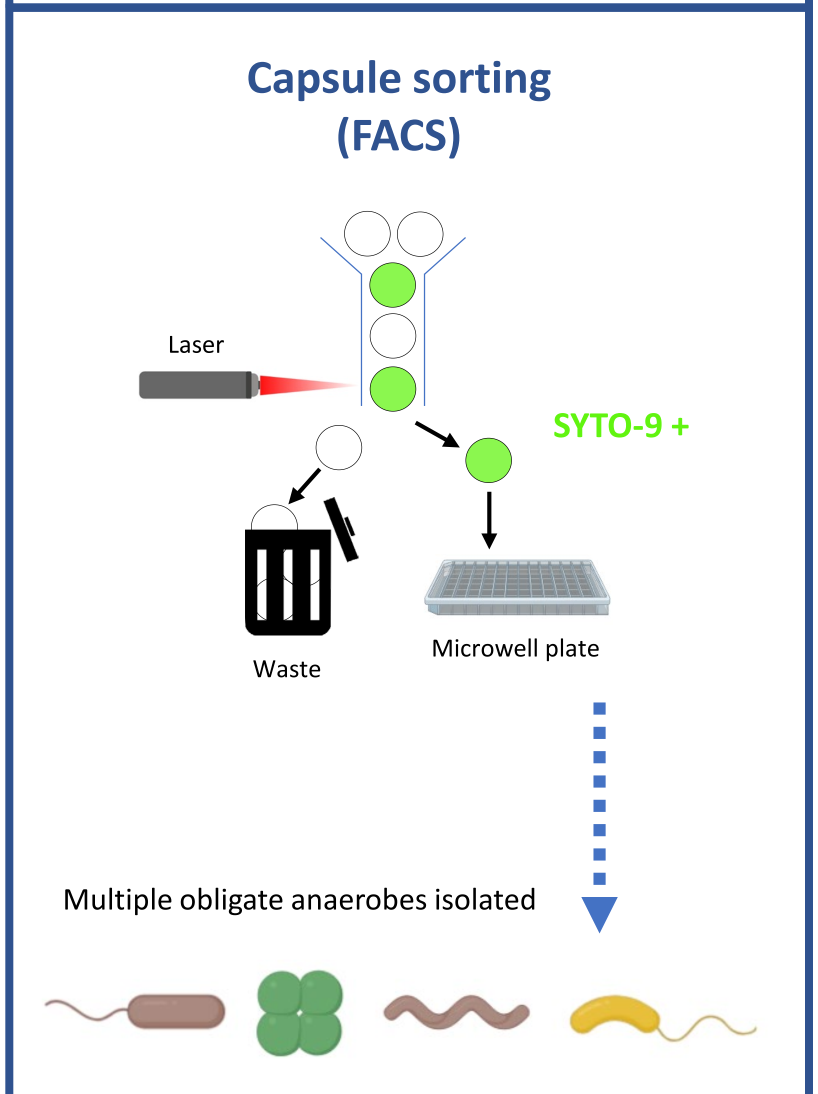

After encapsulation and growth, clonal populations can be isolated by sorting capsules (FACS) in microwell plates. The biomass is stained with SYTO-9.
