# Supplementary figures and images for: High-throughput cultivation and isolation of environmental anaerobes using selectively permeable hydrogel capsules
Source: ISME Commun. 2025 Jul 13;5(1):ycaf117. doi: 10.1093/ismeco/ycaf117 (PMC12319321; doi:10.1093/ismeco/ycaf117)

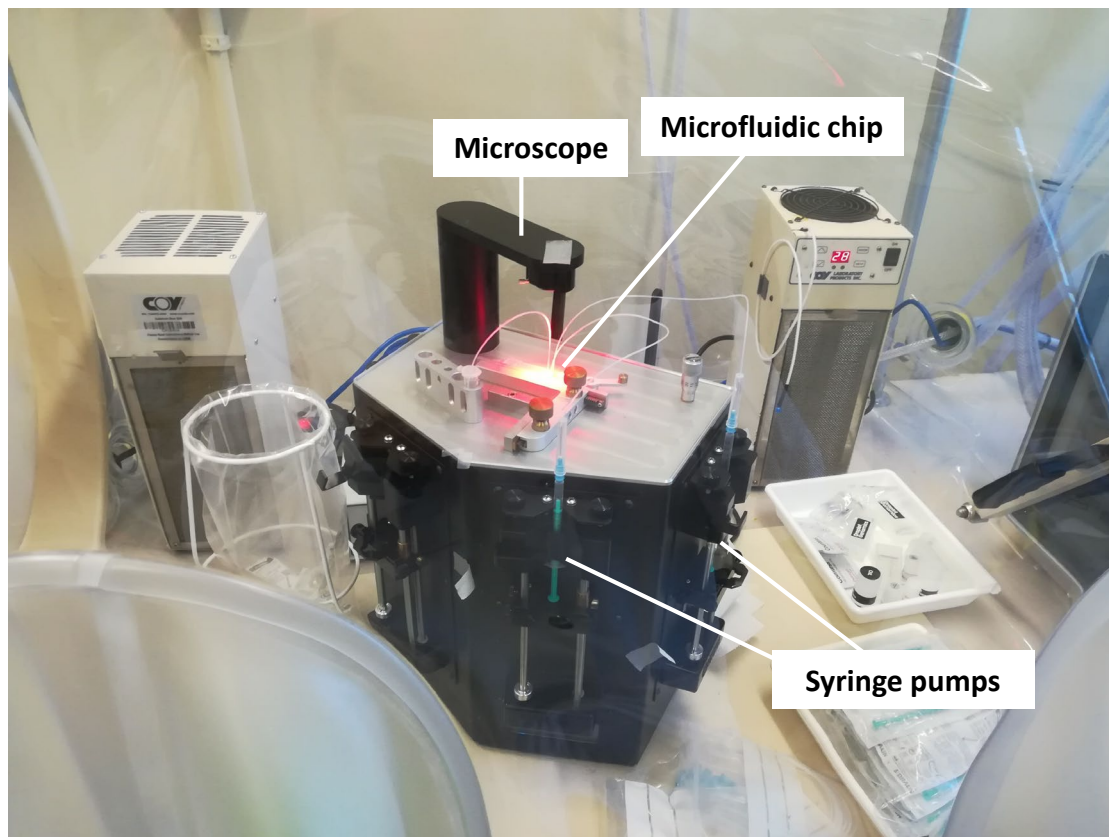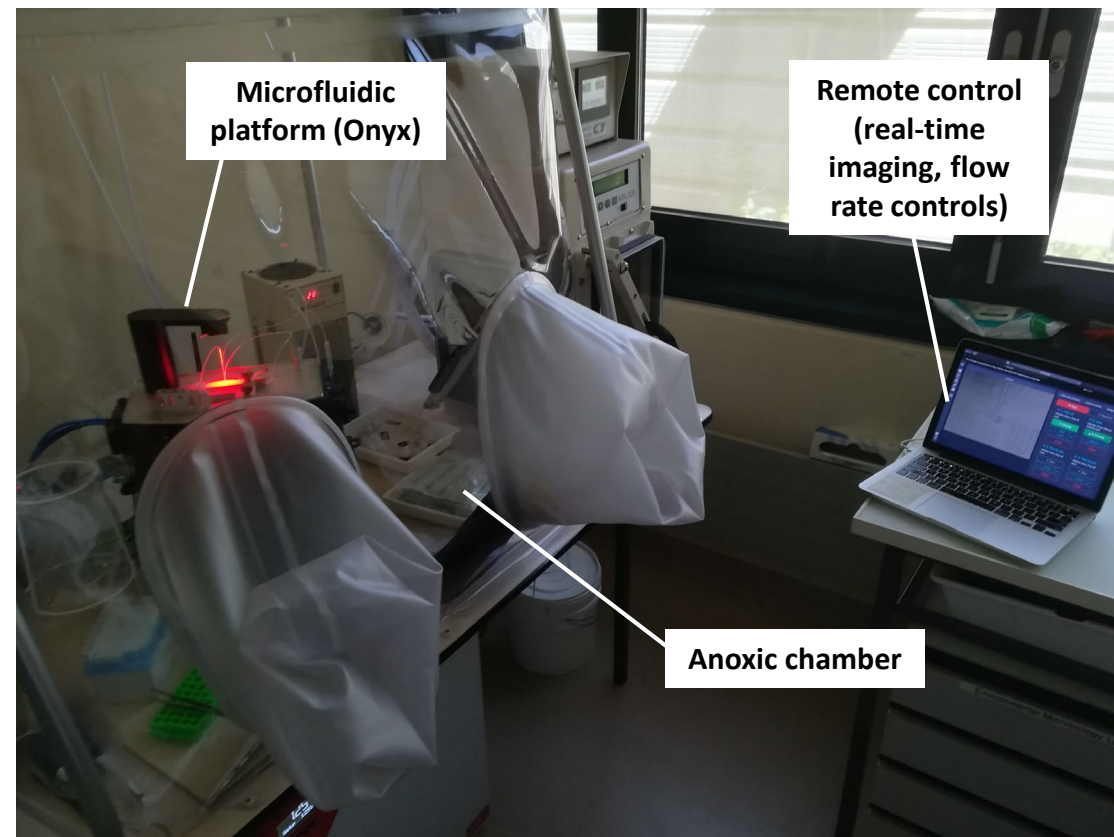

Supplement: figS2_ycaf117 [file figs2_ycaf117.pdf]

Capsules (PBS)

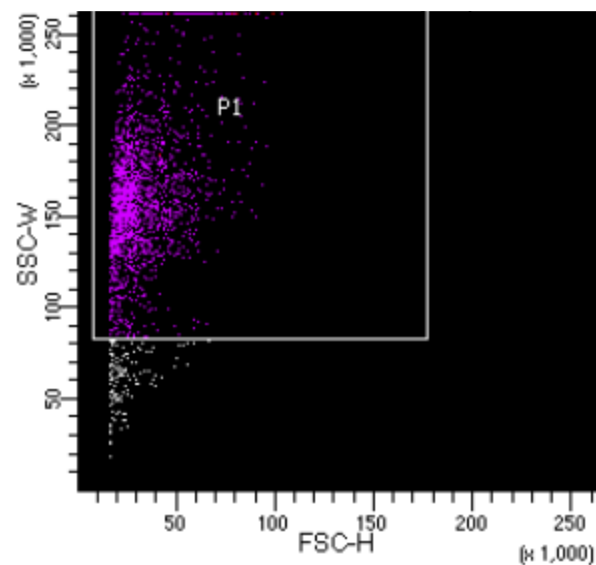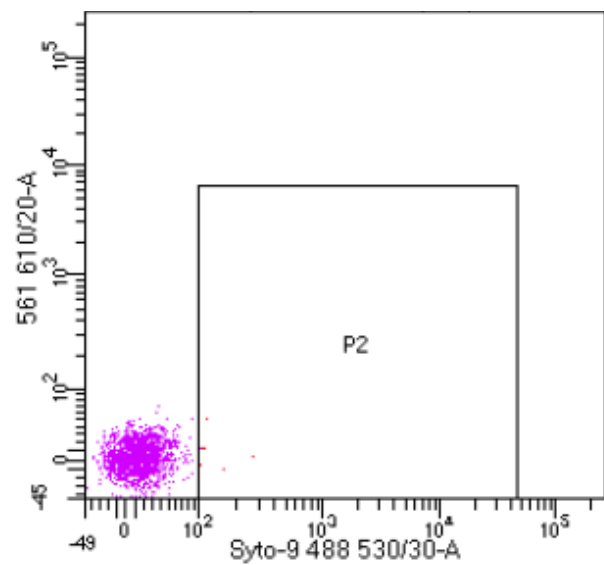

Capsules (medium)

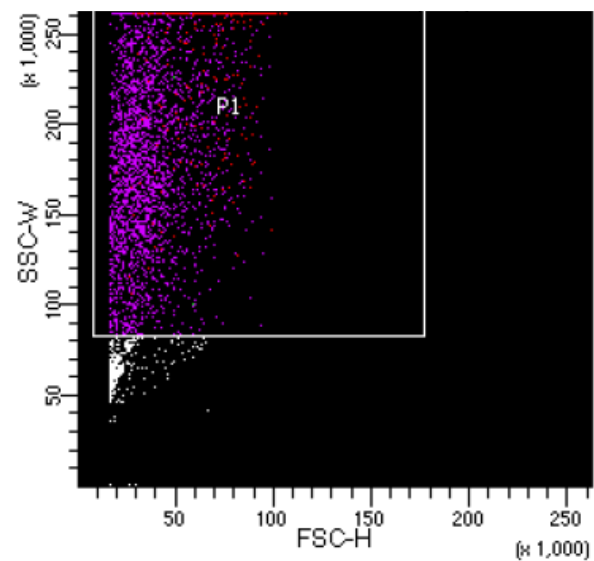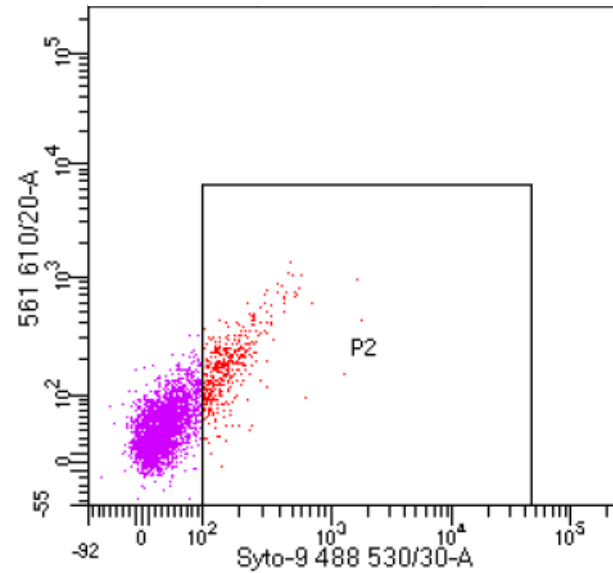

Supplement: figS3_ycaf117 [file figs3_ycaf117.pdf]

*Nitratidesulfovibrio vulgaris*

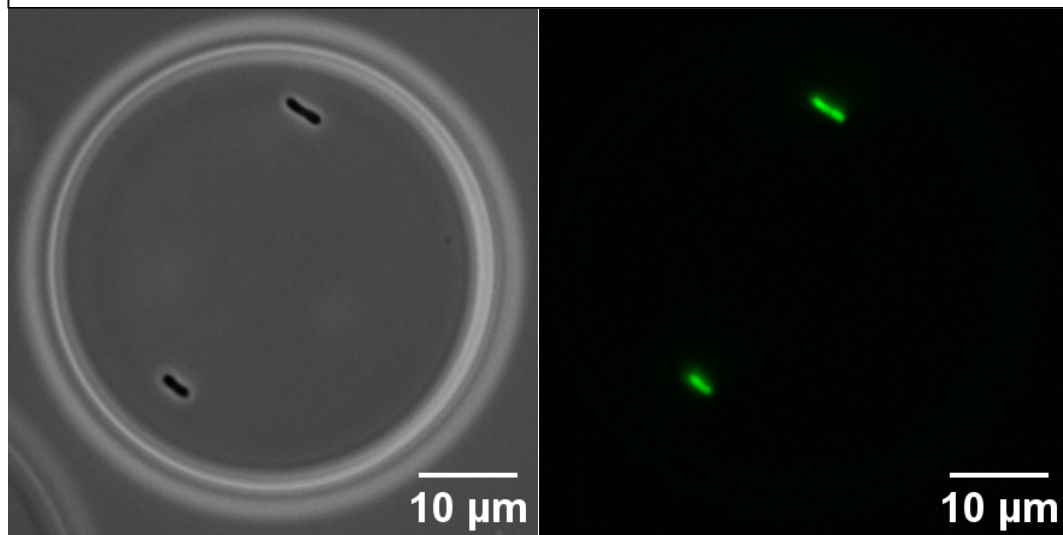

*Shewanella oneidensis* MR-1

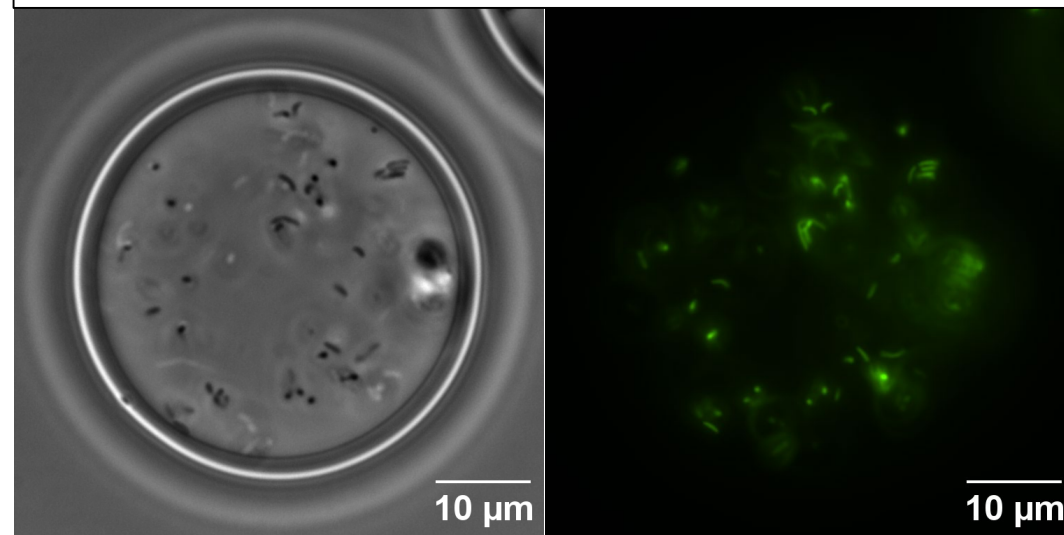

*Escherichia coli* TB205

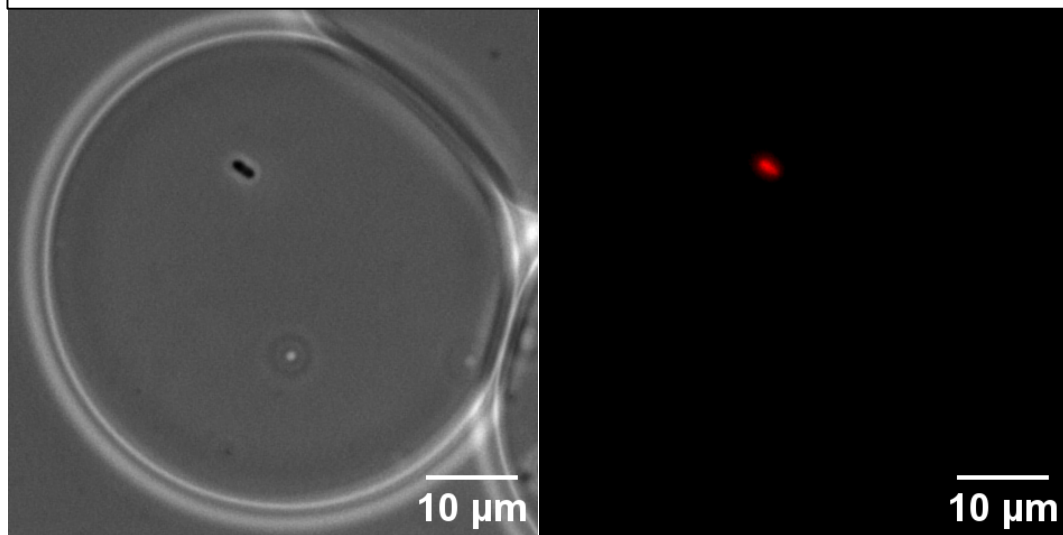

Supplement: figS5_ycaf117 [file figs5_ycaf117.pdf]

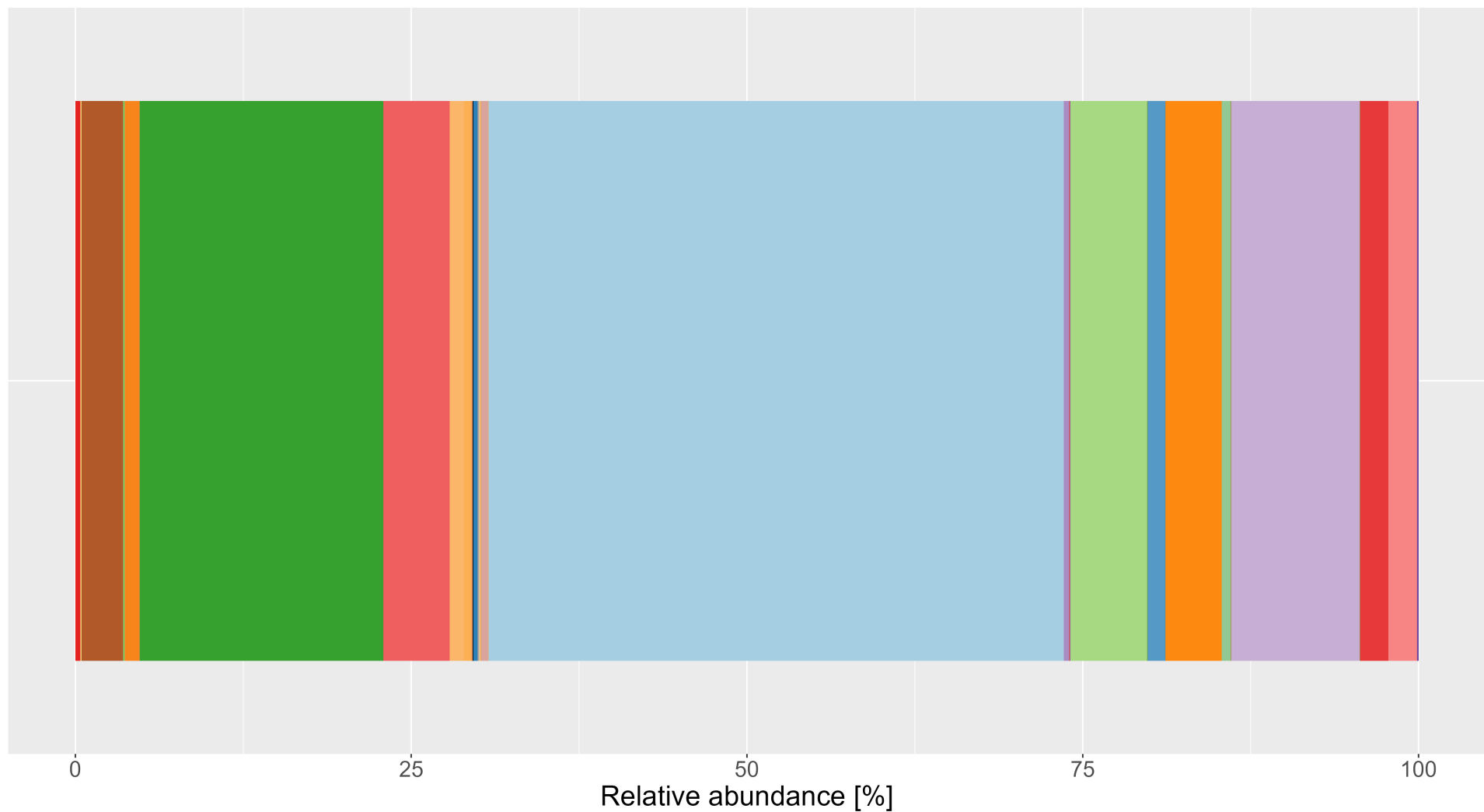

Phylum

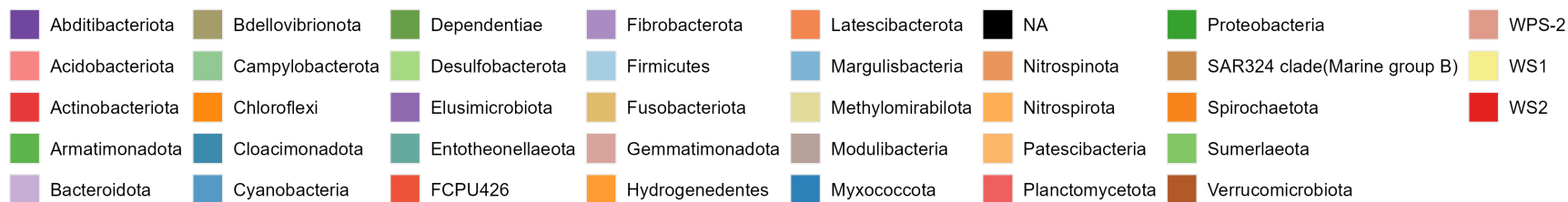

Supplement: figS6_ycaf117 [file figs6_ycaf117.pdf]

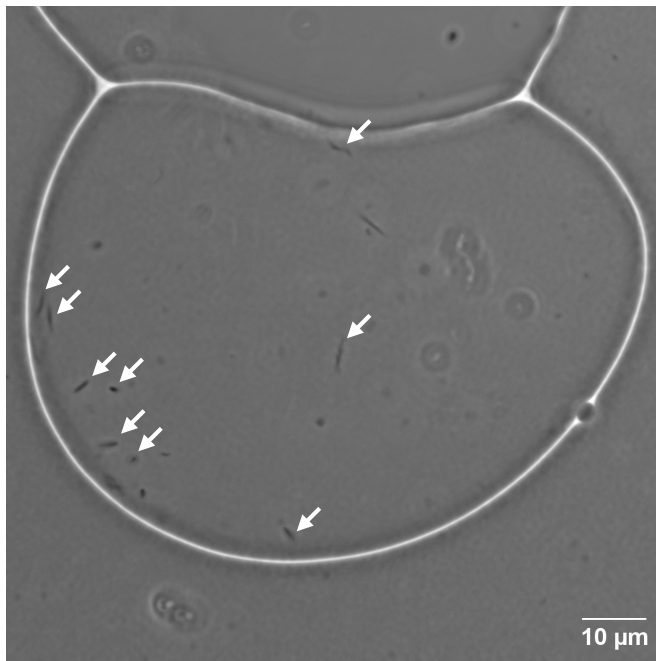

w-o droplets  
15 h

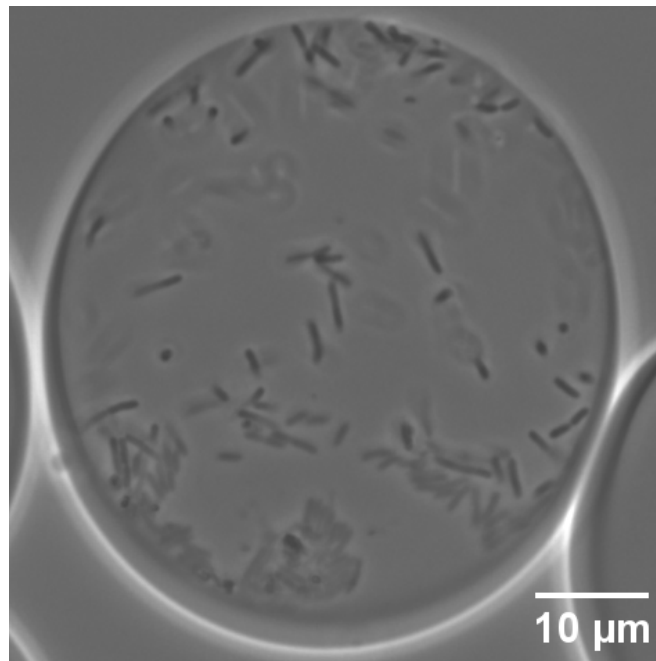

Capsules  
15 h

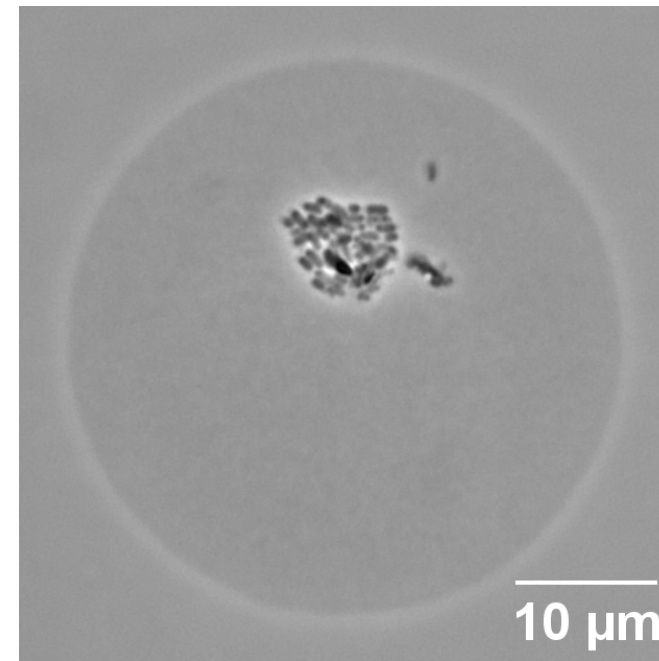

Agarose beads  
4 d

Supplement: figS7_ycaf117 [file figs7_ycaf117.pdf]

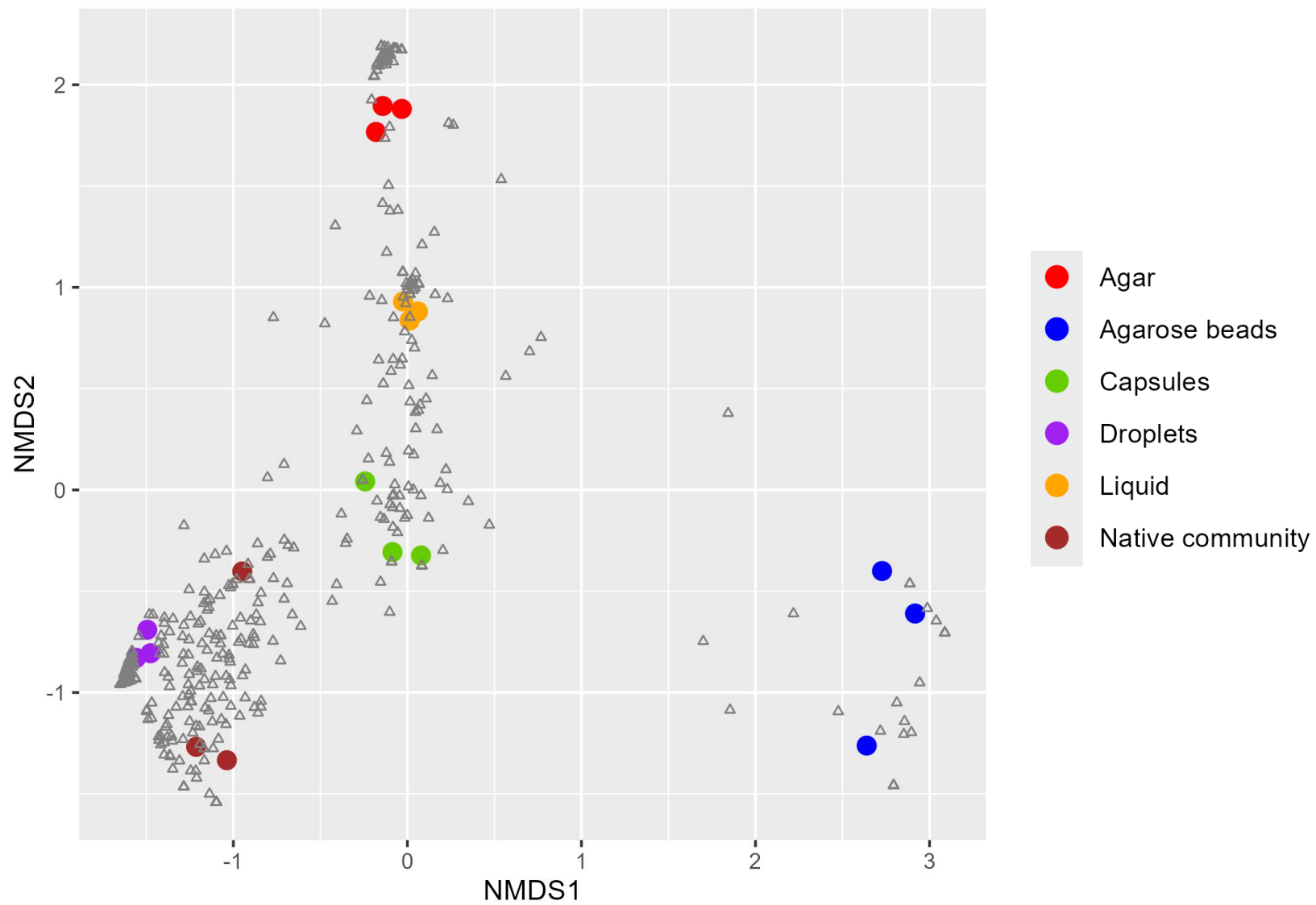

Supplement: figS8_ycaf117 [file figs8_ycaf117.pdf]

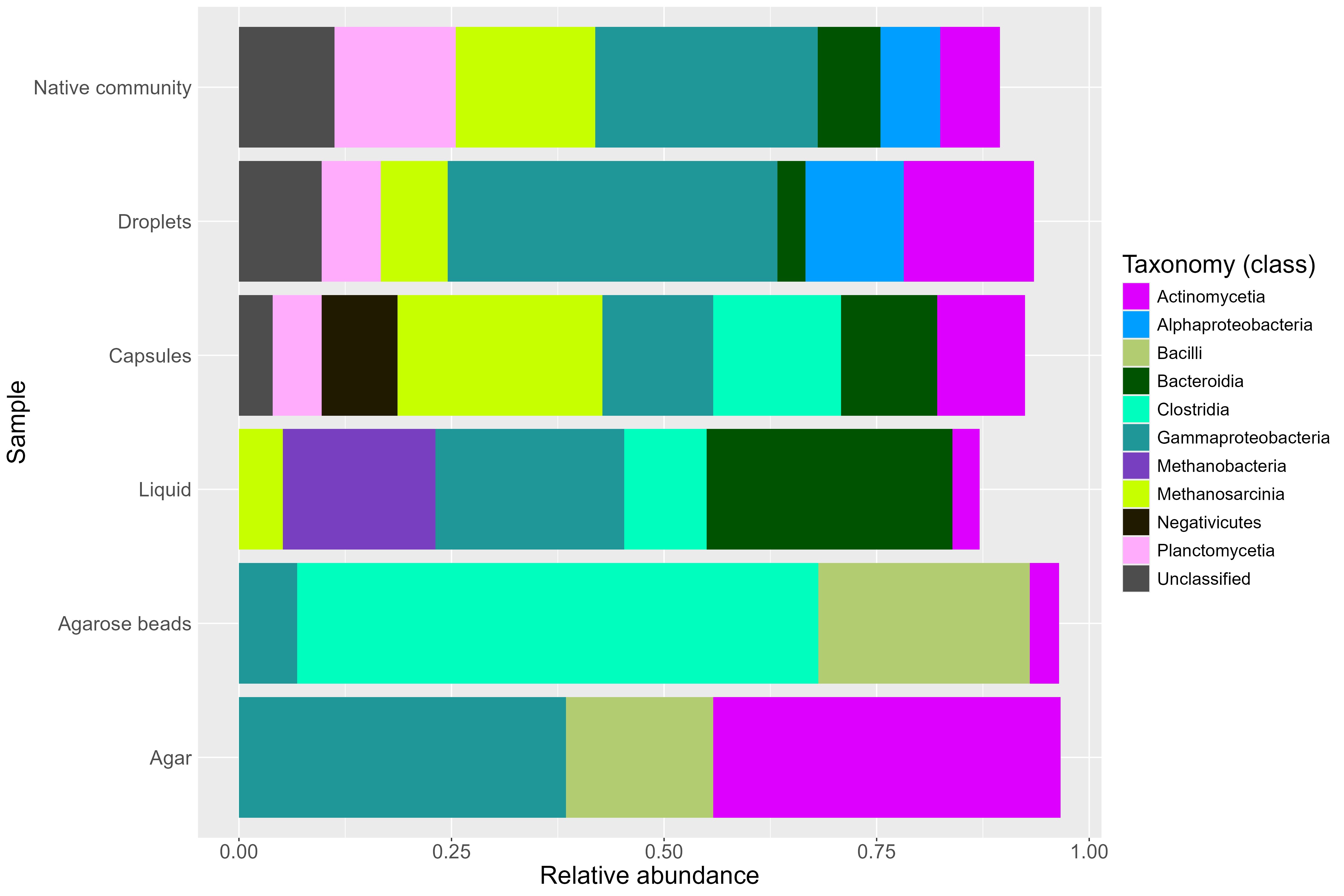

Supplement: figS9_ycaf117 [file figs9_ycaf117.jpeg]

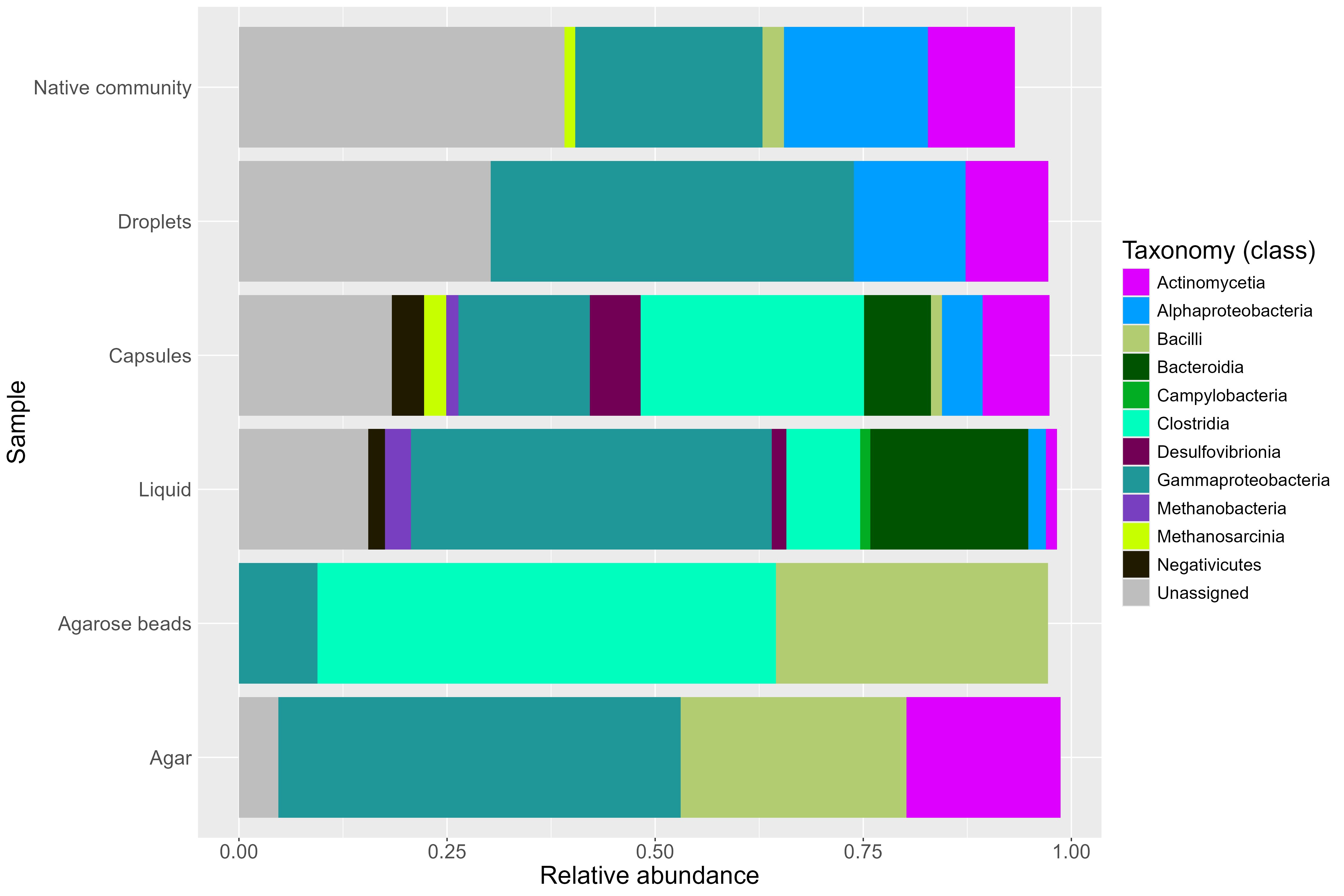

Supplement: figS10_ycaf117 [file figs10_ycaf117.jpeg]

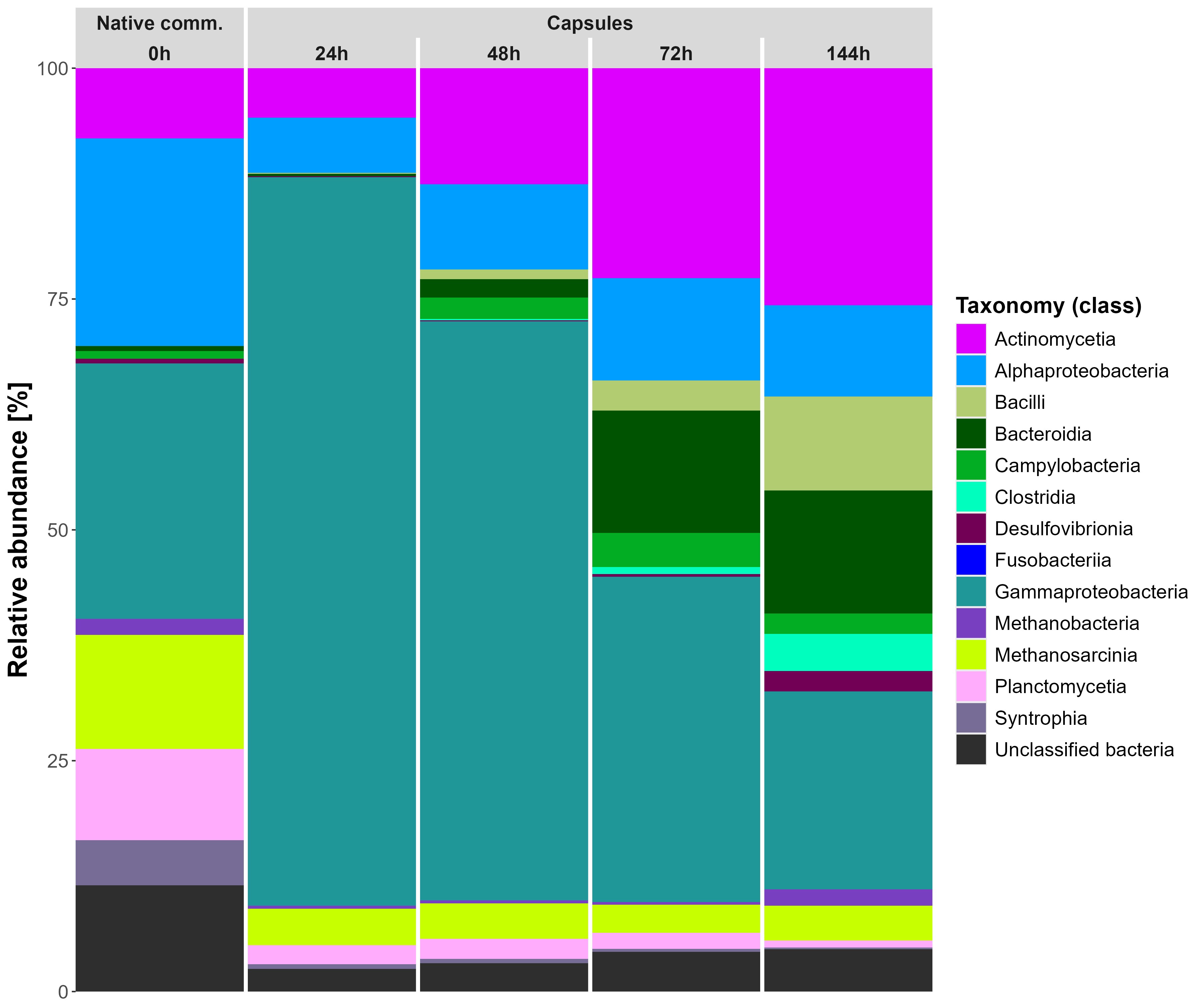

Supplement: figS11_ycaf117 [file figs11_ycaf117.jpeg]

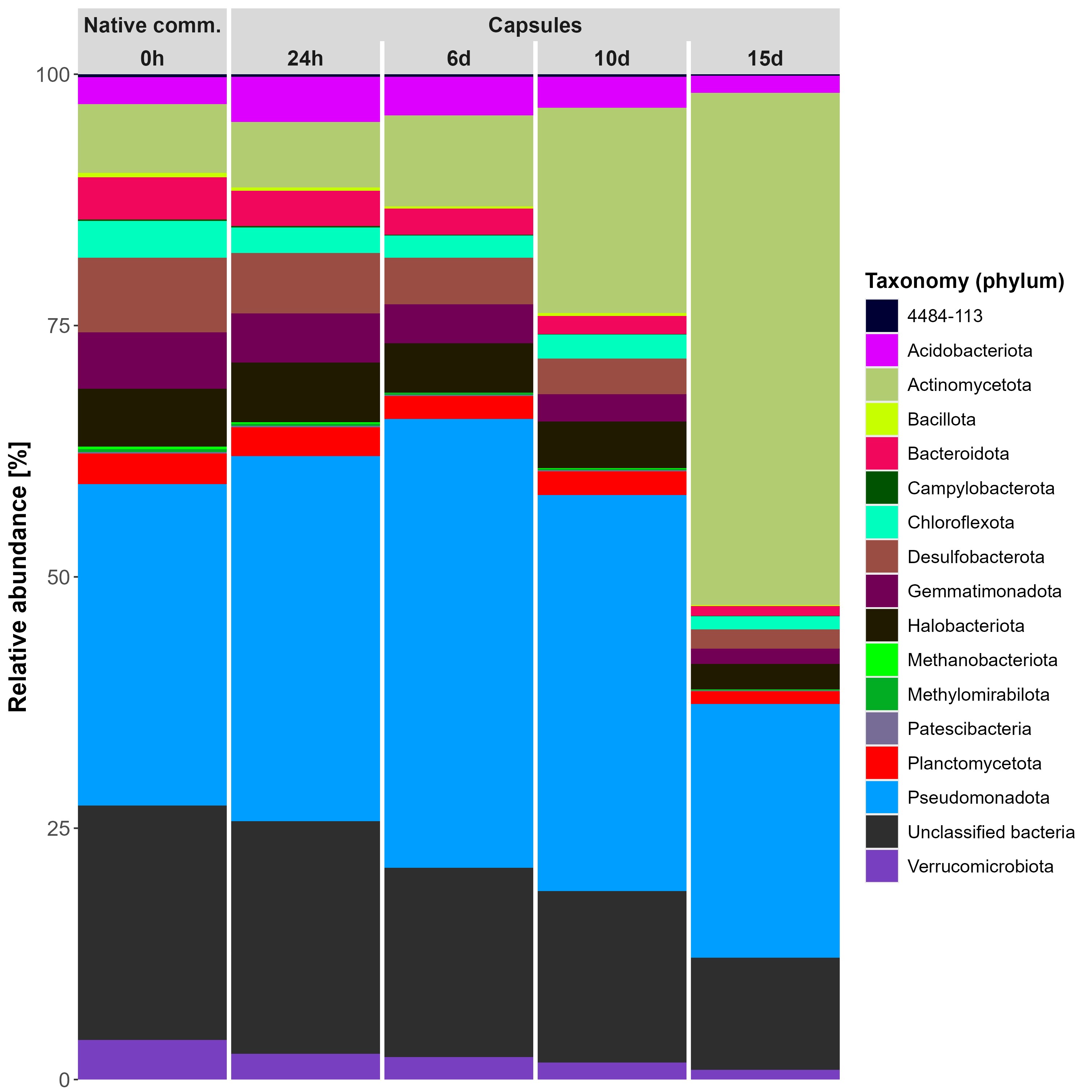

Supplement: figS12_ycaf117 [file figs12_ycaf117.jpeg]

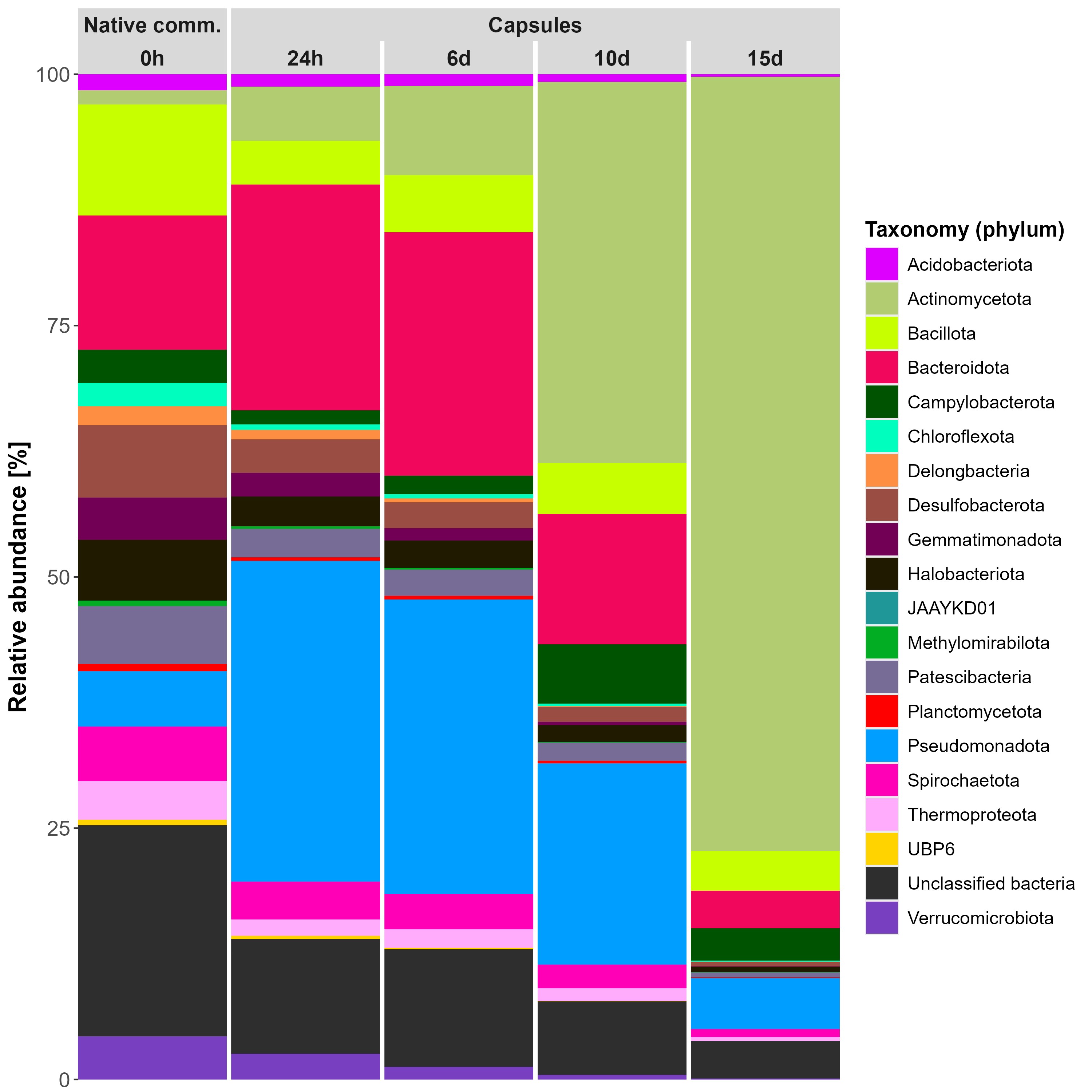

Supplement: figS13_ycaf117 [file figs13_ycaf117.jpeg]

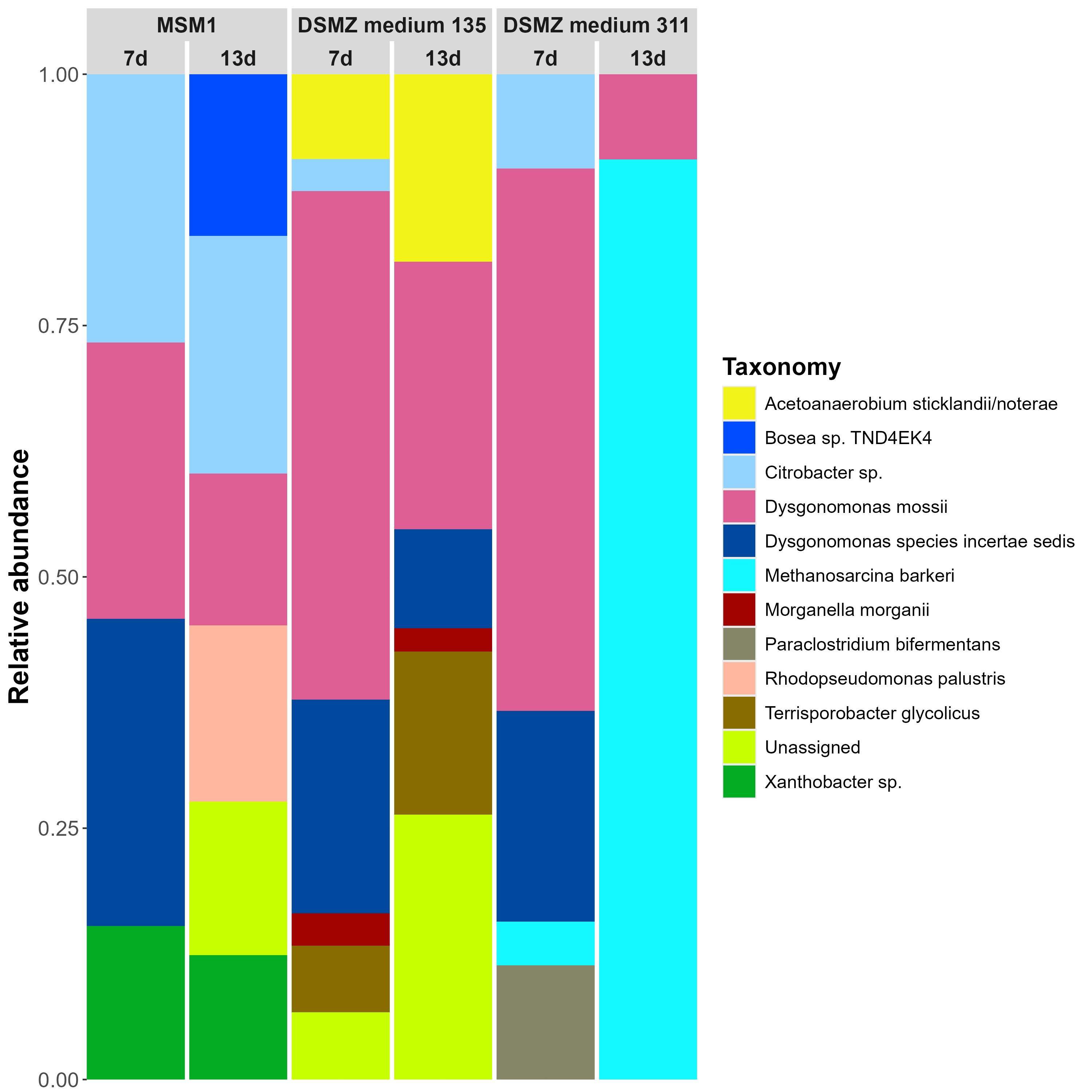

Supplement: figS15_ycaf117 [file figs15_ycaf117.jpeg]

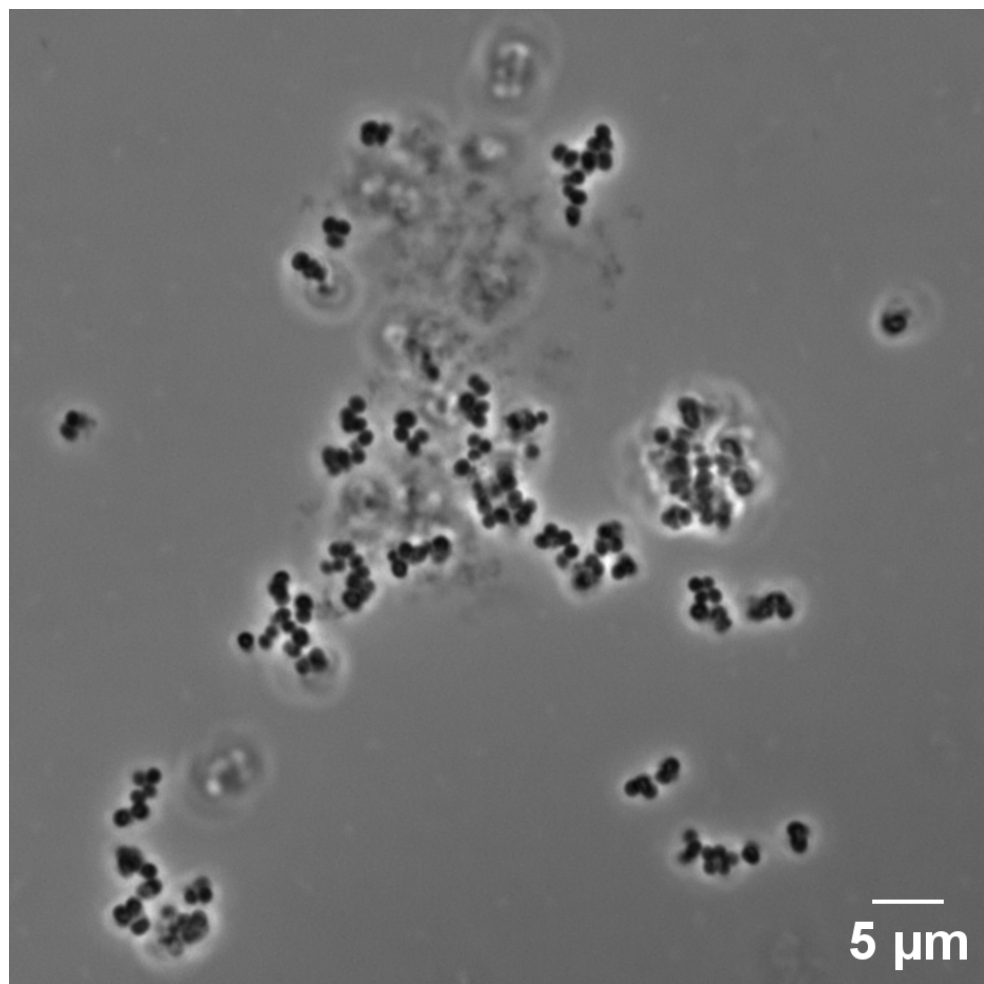

Supplement: figS16_ycaf117 [file figs16_ycaf117.pdf]

Goodness of fit

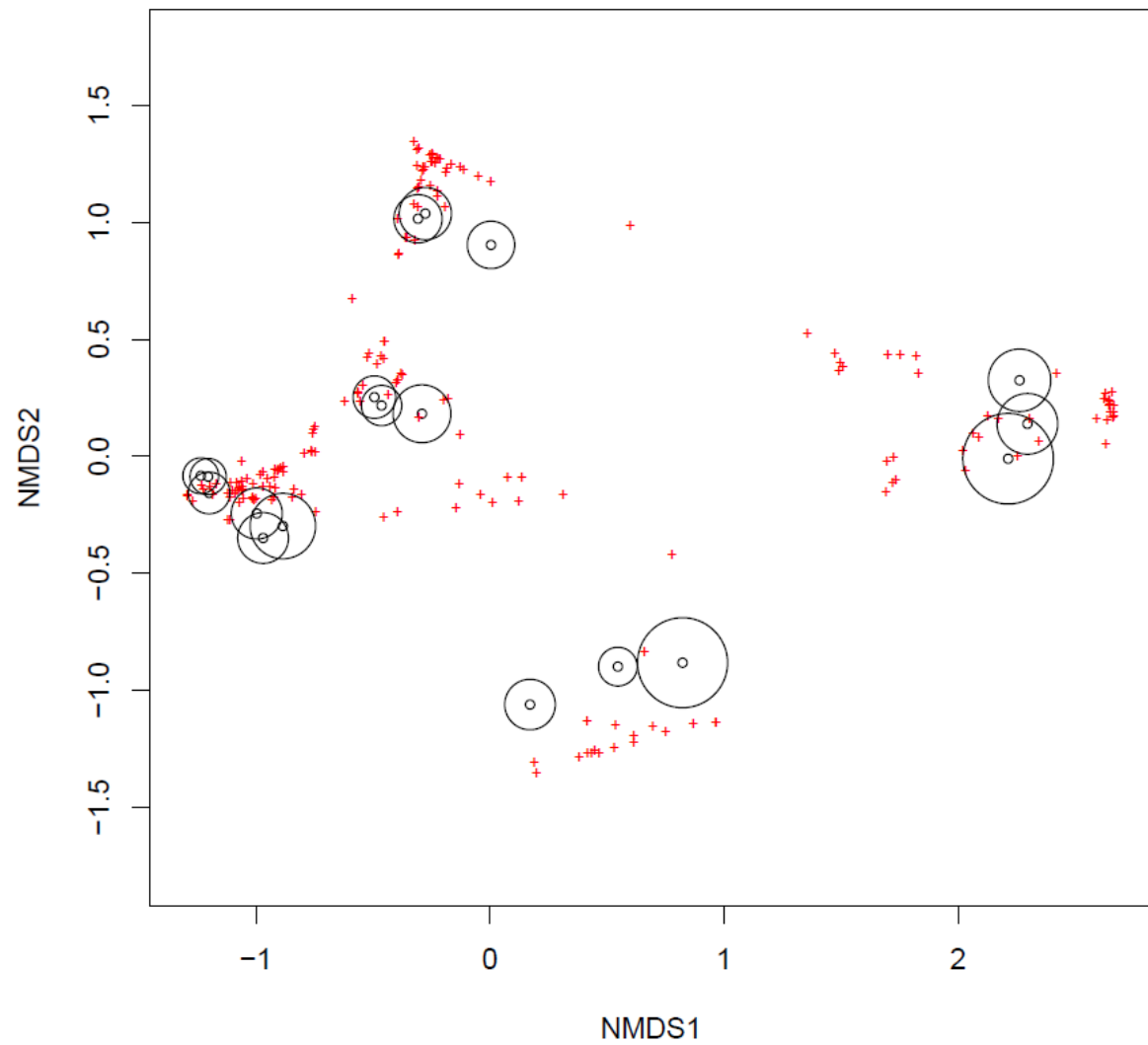

Shepard plot

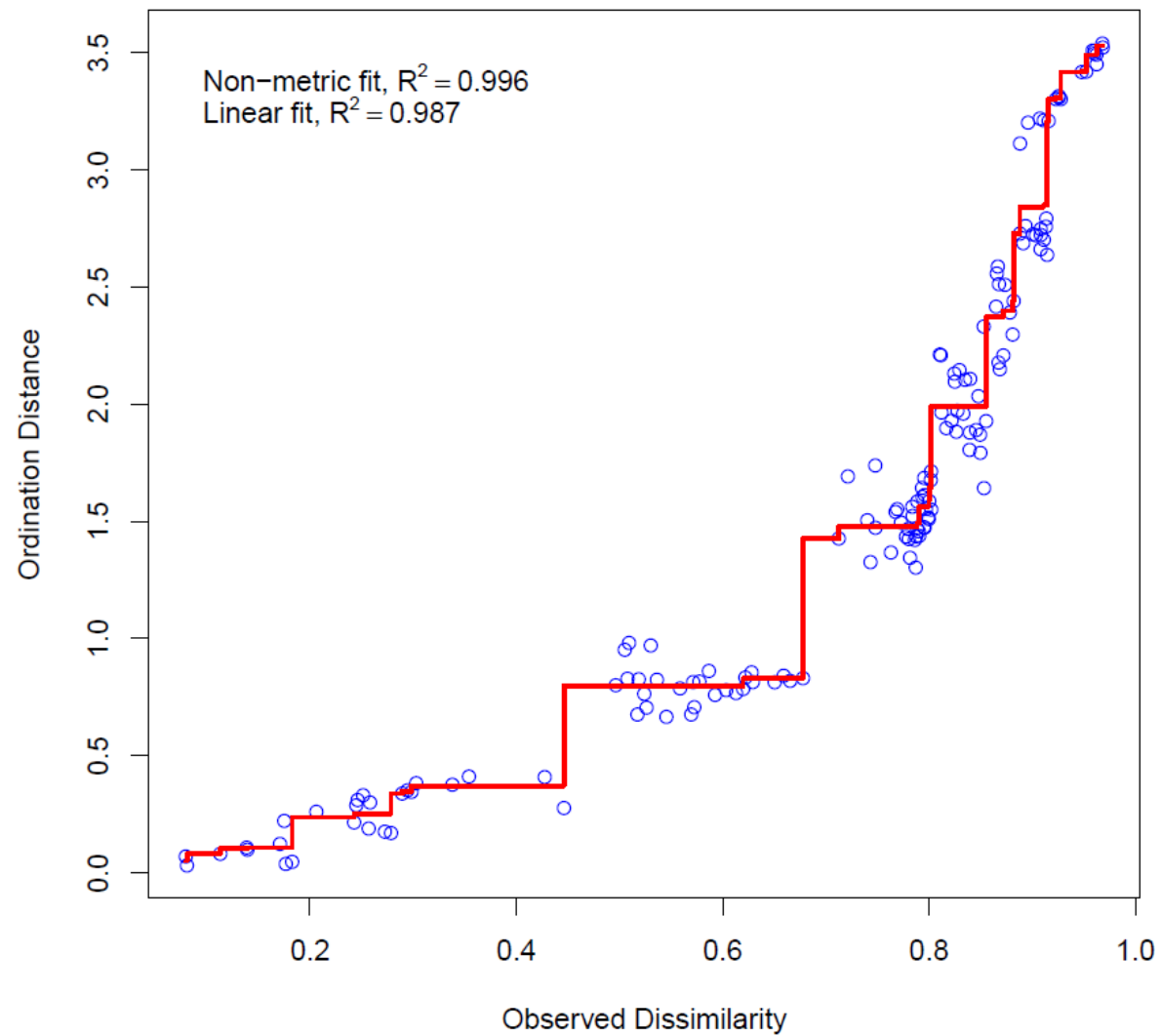

Supplement: figS17_ycaf117 [file figs17_ycaf117.pdf]

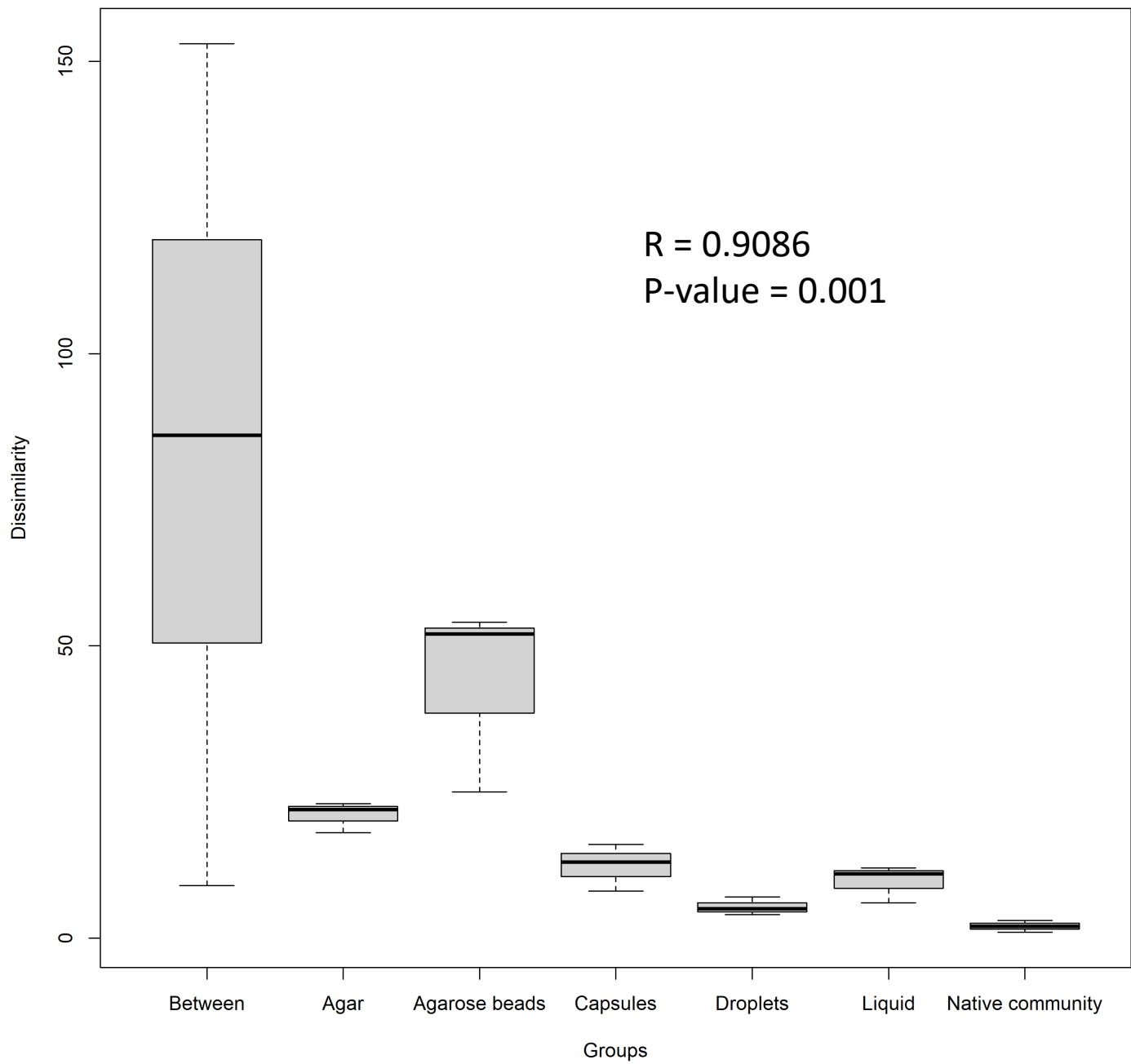

Supplement: figS18_ycaf117 [file figs18_ycaf117.pdf]
