# Supplementary material for: High-throughput cultivation and isolation of environmental anaerobes using selectively permeable hydrogel capsules
Source: ISME Commun. 2025 Jul 13;5(1):ycaf117. doi: 10.1093/ismeco/ycaf117 (PMC12319321; doi:10.1093/ismeco/ycaf117)

Hydrogel capsule

- Permeable
- Liquid compartment
- Planktonic cells
- Compatible with FACS

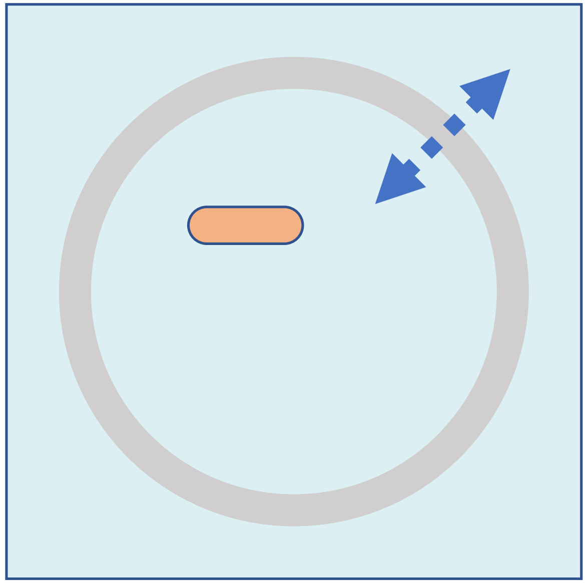

Agarose bead

- Permeable
- Solid compartment (gel matrix)
- Sessile cells
- Compatible with FACS

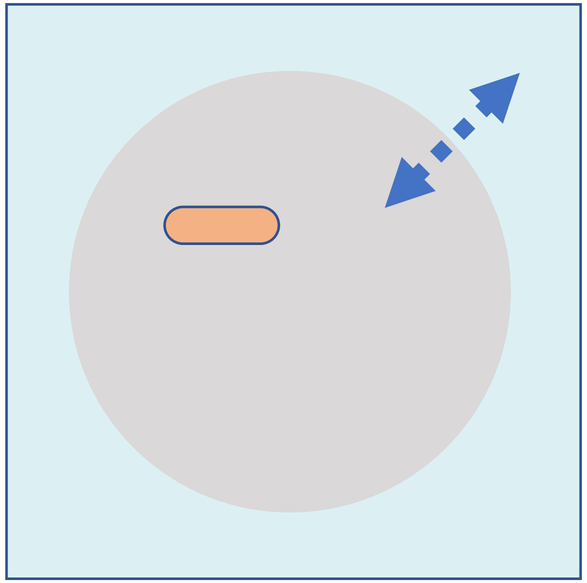

Water-in-oil droplet

- Impermeable (water-oil barrier)
- Liquid compartment
- Planktonic cells
- Not directly compatible with FACS (need double emulsion)

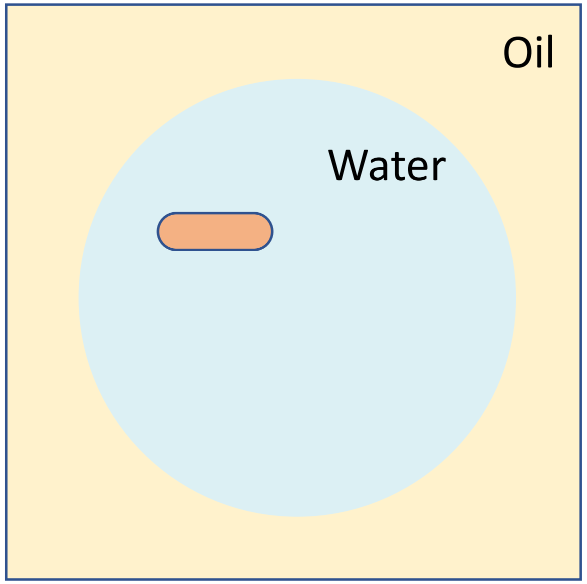

Supplement: figS4_ycaf117 [file figs4_ycaf117.pdf]
