# Supplementary material for: High-throughput cultivation and isolation of environmental anaerobes using selectively permeable hydrogel capsules
Source: ISME Commun. 2025 Jul 13;5(1):ycaf117. doi: 10.1093/ismeco/ycaf117 (PMC12319321; doi:10.1093/ismeco/ycaf117)
Supplement: supplementary_information_and_captions [file supplementary_information_and_captions.pdf]

Supplementary information for:

High-throughput cultivation and isolation of environmental anaerobes using selectively permeable hydrogel capsules

Hugo Sallet, Marion Calvo, Matteo Titus, Nicolas Jacquemin, Karin Lederballe Meibom, Rizlan Bernier-Latmani*

Ecole Polytechnique Federale de Lausanne (EPFL), Environmental Microbiology Laboratory, CH-1015 Lausanne

Corresponding author: Rizlan Bernier-Latmani, EPFL ENAC IIE EML CH A1 375 (Bâtiment CH), Station 6, CH-1015, Lausanne, Switzerland. Email: rizlan.bernier-latmani@epfl.ch

<u>This file contains:</u>

- Supplementary text
- Table S1
- Titles and captions of supplementary movies
- Titles and captions of supplementary figures

Media preparation

All media were brought to a boil, distributed in 200-ml serum bottles (100 ml / bottle) sealed with butyl rubber stoppers and aluminium crimps. The headspace was flushed with anoxic gases before autoclaving at 121°C for 30 min. The composition of the media used in the study can be found below:

Minimal soil medium (MSM): NaCl (1.0 g/l), $MgCl_2 \cdot 6H_2O$ (0.4 g/l), $CaCl_2 \cdot 2H_2O$ (0.1 g/l), NH_4Cl (0.25 g/l), KH_2PO_4 (0.5 g/l), KCl (0.5 g/l), soil extract (10 ml/l for MSM1, 100 ml/l for MSM10), vitamins (cyanocobalamin (50 µg/l), thiamine (100 µg/l), 4-aminobenzoic acid (40 µg/l), D(+)-biotin (10 µg/l), nicotinic acid (100 µg/l), calcium D-(+)-pantothenate (50 µg/l), pyridoxine hydrochloride (150 µg/l)), pH 7.0, CO_2/N_2 (20/80 v/v) headspace. The soil extract was prepared by mixing dry soil (2-mm sieved) with Milli-Q water (1:2 w/w), autoclaving (121°C for 30 min) and filtering the supernatant (0.1 µm). An independent batch of MSM medium was prepared for each soil.

DSMZ 135 medium: NH_4Cl (1 g/l), KH_2PO_4 (0.33 g/l), K_2HPO_4 (0.45 g/l), $MgSO_4 \cdot 7H_2O$ (0.10 g/l), Modified Wolin's mineral solution (20 ml/l), Yeast extract (2 g/l), Sodium resazurin 0.1% w/v (0.5 ml/l), $NaHCO_3$ (10 g/l), D-Fructose (10 g/l), Wolin's 10x vitamin solution (1 ml/l), L-Cysteine $HCl \cdot H_2O$ (0.5 g/l), $Na_2S \cdot 9H_2O$ (0.5 g/l).

DSMZ 311 medium: NH_4Cl (0.5 g/l), $MgSO_4 \cdot 7H_2O$ (0.5 g/l), $CaCl_2 \cdot 2H_2O$ (0.25 g/l), NaCl (2.25 g/l), $FeSO_4 \cdot 7H_2O$ 0.1% w/v in 0.1 N H_2SO_4 (2 ml/l), Trace element solution SL-10 (1 ml/l), Selenite-tungstate solution (1 ml/l), Yeast extract (2 g/l), Casitone (2 g/l), $Betaine \cdot H_2O$ (6.7 g/l), Sodium resazurin 0.1% w/v (0.5 ml/l), K_2HPO_4 (0.35 g/l), KH_2PO_4 (0.23 g/l), Na_2CO_3 (1 g/l), Wolin's vitamin solution (10x) (1 ml/l), L-Cysteine $HCl \cdot H_2O$ (0.3 g/l), $Na_2S \cdot 9H_2O$ (0.3 g/l).

DSMZ 141c medium: KCl (0.34 g/l), $MgCl_2 \cdot 6H_2O$ (4 g/l), $MgSO_4 \cdot 7H_2O$ (3.45 g/l), NH_4Cl (0.25 g/l), $CaCl_2 \cdot 2H_2O$ (0.14 g/l), K_2HPO_4 (0.14 g/l), NaCl (18 g/l), Modified Wolin's mineral solution (10 ml/l), $Fe(NH_4)_2(SO_4)_2 \cdot 6H_2O$ 0.1% w/v (2 ml/l), Na-acetate (1 g/l), Yeast extract (2 g/l), Trypticase peptone (2 g/l), Sodium resazurin 0.1% w/v (0.5 ml/l), $NaHCO_3$ (5 g/l), Wolin's vitamin 10x solution (1 ml/l), L-Cysteine $HCl \cdot H_2O$ (0.5 g/l), $Na_2S \cdot 9H_2O$ (0.5 g/l), Methanol (5 ml/l).

Modified DSMZ 141c medium: same recipe as DSMZ 141c medium, but with the following modifications: amendment of formate (0.3 g/l), no amendment of yeast extract, no amendment of trypticase peptone.

DSMZ medium 63: K_2HPO_4 0.50 g, NH_4Cl 1.00 g, Na_2SO_4 1.00 g, $CaCl_2 \cdot 2H_2O$ 0.10 g, $MgSO_4 \cdot 7H_2O$ 2.00 g, Na-DL-lactate 2.00 g, Yeast extract (1 g/l), Sodium resazurin 0.1% w/v (0.5 ml/l), $FeSO_4 \cdot 7H_2O$ (0.05 g/l), Na-thioglycolate (0.01 g/l), Ascorbic acid (0.01 g/l).

Modified Postgate B medium: as described by Kováč and Kushkevych [1], but with the following modifications: amendment of soil extract (10 ml/l) and sodium resazurin 0.1% w/v (0.5 ml/l).

Modified Wolin's mineral solution: Nitrilotriacetic acid (1.50 g/l), $MgSO_4 \cdot 7H_2O$ (3 g/l), $MnSO_4 \cdot H_2O$ (0.5 g/l), NaCl (1 g/l), $FeSO_4 \cdot 7H_2O$ (0.1 g/l), $CoSO_4 \cdot 7H_2O$ (0.18 g/l), $CaCl_2 \cdot 2H_2O$ (0.1 g/l), $ZnSO_4 \cdot 7H_2O$ (0.18 g/l), $CuSO_4 \cdot 5H_2O$ (0.01 g/l), $AlK(SO_4)_2 \cdot 12H_2O$ (0.02 g/l), H_3BO_3 (0.01 g/l), $Na_2MoO_4 \cdot 2H_2O$ (0.01 g/l), $NiCl_2 \cdot 6H_2O$ (0.03 g/l), $Na_2SeO_3 \cdot 5H_2O$ (3.0×10^{-4} g/l), $Na_2WO_4 \cdot 2H_2O$ (4.0×10^{-4} g/l).

Selenite-tungstate solution: NaOH (0.5 g/l), $Na_2SeO_3 \cdot 5H_2O$ (3 mg/l), $Na_2WO_4 \cdot 2H_2O$ (4 mg/l).

Trace element solution SL-10: HCl (25%) (10 ml/l), $FeCl_2 \cdot 4H_2O$ (1.5 g/l), $ZnCl_2$ (70 mg/l), $MnCl_2 \cdot 4H_2O$ (100 mg/l), H_3BO_3 (6 mg/l), $CoCl_2 \cdot 6H_2O$ (190 mg/l), $CuCl_2 \cdot 2H_2O$ (2 mg/l), $NiCl_2 \cdot 6H_2O$ (24 mg/l), $Na_2MoO_4 \cdot 2H_2O$ (36 mg/l).

Wolin's 10x vitamin solution: Biotin (20 mg/l), Folic acid (20 mg/l), Pyridoxine hydrochloride (100 mg/l), Thiamine HCl (50 mg/l), Riboflavin (50 mg/l), Nicotinic acid (50 mg/l), Calcium D-(+)-Pantothenate (50 mg/l), Vitamin B_{12} (1 mg/l), p-Aminobenzoic acid (50 mg/l), (DL)-alpha-Lipoic acid (50 mg/l).

Enrichments of SRB and methanogens prior to encapsulation

SRB enrichment: 1 g of B_{BH} soil was inoculated in 10 ml modified Postgate B medium in a 25-ml Balch-type tube and incubated at 30°C. Consumption of sulfate and lactate was monitored with ion chromatography (Integrion HPIC, Thermo Fisher Scientific). After 10 days of incubation, the culture was encapsulated.

Methanogen enrichment: 1 g of B_{BH} soil was inoculated in 10 ml modified DSMZ 141c medium in a 25-ml Balch-type tube and incubated at 30°C. Consumption of H_2 and CO_2, as well as methane production, were confirmed by gas chromatography (456-GC, Scion Instruments). After 10 days, the culture was transferred to fresh medium (10% v/v inoculum) and the new culture was encapsulated after 8 days of incubation.

Bioinformatic analysis

Bins were created with multiple binners (MaxBin2, CONCOCT, and MetaBAT2) and different sets of parameters and then dereplicated as described in BASALT: BASALT dereplicates bins through a multi-step process. First, it merges hybrid bins from multiple binning tools and groups similar bins based on ANI ≥ 99% and AF ≥ 50%. It then identifies core sequences using coverage statistics and applies depth normalization to refine bin comparisons. A neural network distinguishing between true biological variation and redundant assemblies based on coverage differences (ΔC) to classify bins as redundant or distinct. Further outlier removal leverages tetranucleotide frequency (TNF) and coverage correlation (CCC) to eliminate contamination. Finally, gap filling with restrained Overlap–Layout–Consensus (rOLC) and reassembly ensures high-quality, non-redundant bins.

MAGs were phylogenetically placed using GToTree (v1.8.4) with the 25 bacterial and archaeal marker genes HMM models. Functional annotation was performed using METABOLIC-G (v4.0) to identify metabolic pathways, while CoverM (v0.7.0) provided MAG abundance across samples after mapping with Strobealign (v0.12.0). The presence of a KEGG module was defined by the presence of 75% of its module steps.

MAGs were labelled depending on genome annotation. MAGs were assigned to aerobic taxa (i.e., those capable of oxygen respiration) based on the presence of gene sets involved in the biosynthesis of cytochrome bc1 complex, cytochrome bd ubiquinol oxidase or cytochrome c oxidase, i.e., with any of the following KEGG modules present: M00151, M00152, M00153, M00154, M00155 or M00156. Those harbouring the genes involved in module M00496 (for reduction of sulfate to APS, reduction of APS to sulfite, and reduction of sulfite to sulfide) in the KEGG database were labelled as SRB. Those harbouring genes for any of the metabolic modules for methanogenesis (hydrogenotrophic, M00567; methylotrophic, M00564 or M00356; acetoclastic, M00357) were labelled as methanogens. MAGs comprising at least 5 of the 7 enzyme-encoding genes involved in the Wood-Ljungdahl pathway (module M00377) were labelled as acetogens.

In some instances, high-quality reads were used for mOTUs profiling with the default database (nr3.0.3) [2], in addition to the taxonomic classification with the GTDB-Tk database.

Richness was defined as the number of dereplicated MAGs (across all samples) with detectable read alignment in a given sample. MAGs were first dereplicated using BASALT (https://github.com/EMBL-PKU/BASALT) to ensure a non-redundant set. Reads were then aligned to this set using Strobealign (https://github.com/ksahlin/strobealign) with default parameters, and abundance estimates were computed using CoverM (https://github.com/wwood/CoverM), also with default settings. No abundance or coverage thresholds were applied; all MAGs with non-zero coverage were included in the richness calculation.

NMDS plots were produced with the metaMDS function (vegan package, R). Goodness of fit and Shepard plots were generated to check the validity and quality of the ordination (Fig. S17), while ANOSIM was used to confirm significance of the differences between groups (Fig. S18). Statistical analyses were performed using the Vegan package in R.

All analyses were conducted on the EPFL high-performance computing (HPC) cluster, utilizing SLURM (v23.11.10) and Apptainer (v1.2.5). Each node consisted of two Intel(R) Xeon(R) Platinum 8360Y processors running at 2.4 GHz, with 36 cores per processor (72 cores per node) and 3 TB of SSD storage.

FACS operating parameters

When sorting capsule, the sorter was equipped with a 100-µm nozzle. Sorting was performed at a frequency of 30 kHz, a pressure of 20 psi and an event rate of 200 s^{-1}.

References

1. Kováč J, Kushkevych I. New modification of cultivation medium for isolation and growth of intestinal sulfate-reducing bacteria. 2017.

2. Ruscheweyh HJ, Milanese A, Paoli L *et al.* Cultivation-independent genomes greatly expand taxonomic-profiling capabilities of mOTUs across various environments. *Microbiome* 2022;**10**:1–12.

Table S1: **soil physical and chemical parameters**

Soil	A_{AH}	B_{AH}	A_{BH}	B_{BH}
Clay content (%)	25.01	26.64	20.96	26.67
Silt content (%)	38.15	57.93	42.20	39.83
Sand content (%)	36.84	15.42	36.84	33.50
Texture	loam	silt loam	loam	loam
pH	7.92	7.49	-	-
TOC (wt %)	2.99	7.71	-	-
C (wt.%)*	4.78 ± 0.04	8.04 ± 0.07	-	-
H (wt.%) *	0.692 ± 0.00	1.32 ± 0.01	-	-
N (wt.%)*	0.25 ± 0.00	0.6 ± 0.01	-	-
S (wt.%)*	0.070 ± 0.02	0.10 ± 0.00	-	-
Ca [mg/kg]	50036	19427	-	-
Fe [mg/kg]	17960	28707	-	-
K [mg/kg]	9116	11863	-	-
Mg [mg/kg]	7473	9779	-	-
Mn [mg/kg]	<loq**	130	-	-
Na [mg/kg]	2168	1906	-	-
P [mg/kg]	<loq**	801	-	-
As [mg/kg]	0.629	0.893	-	-

*Results are indicated as means of three analyses ± standard deviation.

**limit of quantification (loq) = 0.5 mg/l (ICP-OES).

Titles and captions of supplementary movies

Movie 1

Microfluidic generation of hydrogel capsules. The time lapse shows the junction in the microfluidic chip where the aqueous phase (mix of two polymer components) meets the oil phase, resulting in an emulsion.

Movie 2

Time lapse of *Nitratidesulfovibrio vulgaris* growing within hydrogel capsules (2-day incubation).

Movie 3

Time lapse of *E. coli* TB205 growing within hydrogel capsules (24 h incubation).

Movie 4

Time lapse of *Paraclostridium bifermentans* EML growing within hydrogel capsules (24 h incubation).

Movie 5

Time lapse of *Thermoanaerobacter kivui* growing within hydrogel capsules (24 h incubation).

Movie 6

Time lapse of *Shewanella oneidensis* MR-1 growing within hydrogel capsules (24 h incubation).

Titles and captions of supplementary figures

Figure S1: **Graphical summary of the study.**

Figure S2: **Experimental setup used to produce capsules under anoxic conditions**. Left: Capsules are generated with an Onyx microfluidic platform (Atrandi Biosciences) which includes syringe pumps and a microscope. Core and shell reagents (along with the microbial cells) are injected in a microfluidic chip, and the resulting emulsion (which will result in capsules) is collected in an microtube. Right: Overall view of the setup, where capsules are produced inside an anoxic chamber with the Onyx platform, while images and flow rates are monitored on an external device connected through Wi-Fi.

Figure S3: **FACS scatter plots with gates on microcolony-containing capsules.** A first gate (P1) was defined to exclude smaller particles (e.g. free-floating cells or debris), whereas the second gate (P2) further selects the capsules with high SYTO-9 fluorescence signal prior to sorting. Left: analysis of capsules with no microbial growth (incubation in PBS). Right: analysis of capsules with microbial growth (incubation in medium).

Figure S4: **Description of different types of microcompartments.**

Figure S5: **Microscopy images of microorganisms trapped in capsules.** *D. vulgaris* and *E. coli* TB205 were imaged immediately after encapsulation and emulsion breaking (no incubation) whereas *Shewanella oneidensis* MR-1 was imaged after a 24h incubation period in M9 medium at 30°C (see Table 1 for details). For each microbial strain, brightfield (left) and fluorescent (right) images were captured with a microscope (Eclipse Ni-E, Nikon) and disposed side by side. The microbial cells were stained by incubating the sample with SYTO-9 (1 µM) for 10 min in the dark, except for *E. coli* TB205 which constitutively expresses a gene to produce the fluorescent protein mCherry, and thus did not require staining.

Figure S6: **Taxonomic composition of the microbial community in soil A_{AH}.** The taxonomy barplot shows the relative abundance values of indicated microbial taxa at the phylum level. Taxonomy assignment was conducted based on the full-length 16S rRNA genes using the SILVA database.

Figure S7: **Growth of soil microorganisms within microcompartments in a soil-derived minimal medium.** After extraction from soil, single cells were trapped in w-o droplets, capsules and agarose beads and incubated at 30°C in MSM10. Images taken after different times reveal the growth of organisms in droplets (left), capsules (middle) and agarose beads (right). Arrows in the left figure point to microbial cells.

Figure S8: **Nonmetric multidimensional scaling (NMDS) plot illustrating differences across microbial communities, based on Bray-Curtis dissimilarity values derived from relative abundance data (mOTUs).** mOTUs reads which did not map to any known reference were removed from the analysis and relative abundance values were rescaled accordingly. Each OTU is shown as a triangle, whereas communities are depicted as colours dots. Different colours indicate different cultivation conditions (three replicates per condition). The stress value of the ordination was 0.0589.

Figure S9: **Taxonomy barplot of the soil microbial communities across different cultivation platforms (MAGs).** Relative abundance values are shown as means of three replicates. Taxonomy is displayed at the class level. MAGs with relative abundance >3% are displayed. Reads which do not map to any MAG are not shown. The 'Unclassified' label indicates MAGs which map to bacteria of unknown phyla.

Figure S10: **Taxonomy barplot of the soil microbial communities across different cultivation platforms (mOTUs).** Relative abundance values are shown as means of three replicates. Taxonomy is displayed at the class level. OTUs with relative abundance >1% are displayed. The 'Unassigned' label indicates OTUs which do not map to any reference in the mOTUs database.

Figure S11: **Temporal dynamics of microbial communities (soil A_{BH}) growing within capsules in medium replenished after each sampling.** Taxonomy barplot showing changes in the microbial community throughout cultivation. After each time point, the old medium was replaced with fresh medium. The MAGs are shown at the class level (see colour code in legend). Taxa labelled as 'Unclassified bacteria' are MAGs that were not assigned to a known phylum.

Figure S12: **Temporal dynamics of microbial communities growing within capsules (soil A_{BH}, MSM1 medium) over a longer time period.** Taxonomy barplot showing changes in the microbial community throughout cultivation. Relative abundance values are shown as means of three replicates. MAGs are shown at the phylum level (see colour code in legend). Taxa labelled as 'Unclassified bacteria' are MAGs that were not assigned to a known phylum.

Figure S13: **Temporal dynamics of microbial communities growing within capsules (soil B_{BH}, MSM1 medium) over a longer time period.** Taxonomy barplot showing changes in the microbial community throughout cultivation. Relative abundance values are shown as means of three replicates. MAGs are shown at the phylum level (see colour code in legend). Taxa labelled as 'Unclassified bacteria' are MAGs that were not assigned to a known phylum.

Figure S14: **Pie charts of all isolates obtained across experiments listed in Table 2.** Each chart corresponds to a separate experiment. The isolates listed in Table 2 are highlighted as exploded slices, with genus-level taxonomy displayed.

Figure S15: **Taxonomy barplots showing the changes in relative abundance of capsule-grown microorganisms (derived from soil A_{BH}) after 7 and 13 days of incubation in different media.** The taxonomy was profiled with mOTUs.

Figure S16: **Microscopy image of *Methanococcus maripaludis* MM901 cells growing in the microwell plate after FACS.** Single-cell encapsulation was performed on a pure culture of *Methanococcus maripaludis* MM901. The capsules were incubated in DSMZ 141c medium for 1 month after which single capsules were sorted and distributed in a 96-well plate filled with DSMZ 141c medium. Immediately after sorting, the 96-well plate was incubated in an anaerobic jar filled with H_2/CO_2 (80/20 v/v) headspace. After one week of incubation, the plate was taken out of the jar, and growth could be seen visually in several wells. The image shown was taken from one of these wells.

Figure S17: **Goodness of fit and Shepard plots for the NMDS.** Left: the goodness of fit of the representation is illustrated for each sample by the diameter of the circles around them (larger circles indicating poorer fits) whereas red crosses indicate MAGs. Right: the Shepard plot shows the agreement (high R-squared values) between the original distance matrix and the ordination representation.

Figure S18: **Analysis of similarities (ANOSIM) plot showing dissimilarity between and within communities in the NMDS ordination.** The bold line within each box represents the median. The lower edge of the box marks the 25th percentile, while the upper edge marks the 75th percentile. Whiskers extend to the most extreme data points within 1.5 times the interquartile range from the box.
